# Optimization of LC-MS^2^ Data Acquisition Parameters for Molecular Networking Applied to Marine Natural Products

**DOI:** 10.3390/metabo12030245

**Published:** 2022-03-14

**Authors:** Sam Afoullouss, Agata Balsam, A. Louise Allcock, Olivier P. Thomas

**Affiliations:** 1 School of Biological and Chemical Sciences, Ryan Institute, National University of Ireland Galway (NUI Galway), University Road, H91TK33 Galway, Ireland; s.afoullouss1@nuigalway.ie (S.A.); a.balsam1@nuigalway.ie (A.B.); 2 School of Natural Sciences, Ryan Institute, National University of Ireland Galway (NUI Galway), University Road, H91TK33 Galway, Ireland; louise.allcock@nuigalway.ie

**Keywords:** MZmine2, GNPS, optimization, data-dependent acquisition, feature-based molecular networking

## Abstract

Since the introduction of the online open-source GNPS, molecular networking has quickly become a widely applied tool in the field of natural products chemistry, with applications from dereplication, genome mining, metabolomics, and visualization of chemical space. Studies have shown that data dependent acquisition (DDA) parameters affect molecular network topology but are limited in the number of parameters studied. With an aim to optimize LC-MS^2^ parameters for integrating GNPS-based molecular networking into our marine natural products workflow, a design of experiment (DOE) was used to screen the significance of the effect that eleven parameters have on both Classical Molecular Networking workflow (CLMN) and the new Feature-Based Molecular Networking workflow (FBMN). Our results indicate that four parameters (concentration, run duration, collision energy and number of precursors per cycle) are the most significant data acquisition parameters affecting the network topology. While concentration and the LC duration were found to be the two most important factors to optimize for CLMN, the number of precursors per cycle and collision energy were also very important factors to optimize for FBMN.

## 1. Introduction

Molecular networking is an informatics tool that allows visualization of non-targeted tandem mass spectrometer data (MS^2^), to highlight structure similarities between metabolites of a complex mixture and help in the annotation of the detected metabolites [1]. The most common data acquisition technique for molecular networking uses data-dependent acquisition (DDA) [2]. DDA is an autonomous data acquisition mode and works by first taking an MS^1^ scan and collecting the *m*/*z* and relative abundance of analytes. This is instantly followed by multiple MS^2^ scans, targeting the major analytes selected from the MS^1^ scan. Molecular networking is now broadly used in the field of natural products (NP) with the introduction of online molecular networking Global Natural Products Social (GNPS) platform, developed by Wang et al. [3] in 2016. GNPS has been applied to a wide range of applications including dereplication [4,5,6,7], metabolomics [8,9,10,11], and genome mining [12,13]. 

GNPS uses an algorithm to compare the similarities of fragmentation spectra (MS^2^) in each dataset, generating a cosine score for each pair of MS^2^ spectra. Working on the principle that structurally similar molecules will produce MS^2^ spectra with fragment ions in common [14], the cosine scores aim to measure spectral similarity. Using the MS-Cluster algorithm [15], MS^2^ spectra with identical parent ion masses are combined to produce a single consensus spectrum and are represented by a single node, characterized by its *m*/*z* and MS^2^ fragment ion patterns. Nodes with similar MS^2^ spectra, and therefore high cosine scores (usually set at more than 0.7), are connected via edges to form a cluster. Clusters can be considered “molecular families”, as the metabolites should share key chemical features. When combined with automated searches of databases containing MS^2^ spectra, the known compounds can quickly be annotated, turning molecular networking and GNPS into a powerful dereplication tool [16]. A key feature of GNPS is the ability for users to generate and share MS^2^ spectra for identified compounds, which can be uploaded to open-access GNPS reference spectra libraries. Thus, the natural products community can contribute to the rapid growth of reference spectra libraries, increasing the range of natural products which can be annotated quickly.

GNPS contains two main workflows to create molecular networks: Classical Molecular Networking (CLMN) and Feature-Based Molecular Networking (FBMN) [17]. Both workflows use the same untargeted LC-MS^2^ data. CLMN was the first tool to be introduced as a quick and effective way to visualize the chemical space of a sample, creating networks using only MS^2^ spectra. The FBMN workflow advances on CLMN by using both MS^1^ (e.g., isotopic pattern, retention time) and MS^2^ data to create more reproducible and accurate molecular networks. In the FBMN workflow, LC-MS^2^ data are processed using MZmine2 [18] or similar. This processing allows FBMN to be used for relative quantification and increases reproducibility including the ability to resolve isomers [17]. Both molecular networking workflows have been widely utilized in natural product related fields [3].

**Figure 1:**
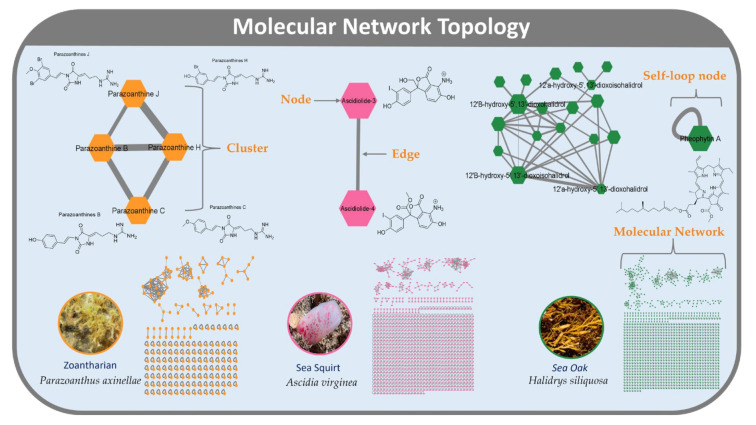
Annotated molecular networks of the studied samples, *Parazoanthus axinellae* (yellow, **left**), *Ascidia virginea* (pink, centre), and *Halidrys siliquosa* (green, **right**), showing the diverse range of metabolites. Elements of molecular networking topology are labelled.

Recent works showed that molecular network topologies are affected by DDA parameters, including intensity threshold, collision energy, and exclusion after n scans [18,19,20]. Each of these studies employed a One Factor at a Time (OFAT) approach and investigated a limited number of parameters. Our preliminary investigation did likewise indicate that the four parameters tested (concentration, liquid chromatography duration, precursors per cycle, and collision energy) had a significant impact on CLMN [21]. We recognized that an OFAT approach, while simple to implement, was inefficient for testing a large range of parameters. This approach also limits the ability to observe interactions between parameters. We therefore used a fractional factorial design to evaluate the effect of multiple LC-ESI-MS^2^ data acquisition parameters on the resulting molecular network. Two key advantages of using the fractional factorial design are: (i) it allows for the statistical significance of each parameter to be determined; (ii) it assesses the significance of interactions between parameters. To cover the diverse range of families of marine natural products with distinct physico-chemical properties, we analysed extracts of three widely differing marine organisms present off the coasts of Ireland: the sea squirt *Ascidia virginea*, rich in small phenolic derivatives currently under chemical investigation, the zoantharian *Parazoanthus axinellae* known to contain a range of polar alkaloids, and the macroalga *Halidrys siliquosa*, rich in meroterpenoids (Figure 1). To the best of our knowledge this is the first study optimizing LC-MS^2^ data acquisition parameters for FBMN. 

## 2. Results and Discussion

### 2.1. Response Models

Of the 36 response models generated using fractional factorial analysis, the majority had high Predicted R^2^ (R^2^ Pred.) and *p*-values < 0.01 (Table S8) indicating a high goodness-of-fit. Residual plots (Figures S4–S39) were also analyzed to evaluate the fit of the models. Deviation in the quality of response models was seen between the two molecular networking workflows and between the three samples. Key nomenclature for this study is summarized in Figure 2.

Models with poor fit (*p*-value > 0.05 or R^2^ Pred. < 20) were not included in the response analysis. Analysis of CLMN significant factors excluded response models for clustering co-efficient, number of neighbors and two of three average cosine models, due to poor model fit. Response models for FBMN produced better quality models. *Parazoanthus axinellae* models were an outlier with response models for the number of nodes, neighbors and average cosine being excluded from further analysis due to poor fit with data. 

### 2.2. Significant Factors and Significant Factor Interactions

**Figure 3:**
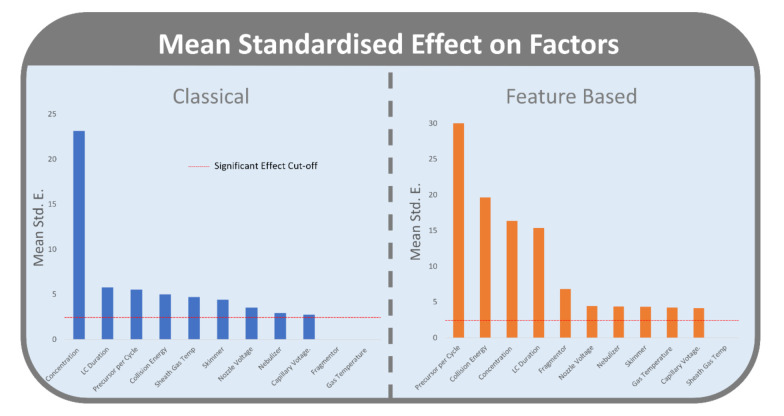
Significant effects of factors on CLMN (blue, **left**) and FBMN (orange, **right**), averaged across response models and samples. For included models, refer to Figure S11. Standardized Effects (Std E) of 2.27 or greater is considered significant (red dashed line).

When averaged across response models and sample, only two factors, fragmentor voltage and drying gas temperature did not have a significant effect on the measured responses in the CLMN workflow. While the effect of all other factors was significant (Figure 3), they varied in the size of their Standardized Effect (Std E.). Sample concentration (Std E. 23.1) had the greatest effect, followed by LC duration (Std E. 5.8), precursor per cycle (Std E. 5.5), collision energy (Std E. 5.0), sheath gas temperature (Std E. 4.7), skimmer voltage (Std E. 4.4), nozzle voltage (Std E. 3.5), nebulizer pressure (Std E. 2.9), and capillary voltage (Std E. 2.7).

In the FBMN workflow, all factors, except sheath gas temperature, had a significant effect on the measured responses. Precursor per cycle (Std E. 33.4) had the greatest standardized effect, followed by collision energy (Std E. 19.6), sample concentration (Std E. 16.4), LC duration (Std E. 15.4), fragmentor voltage (Std E. 6.9), nozzle voltage (Std E. 4.5), nebulizer pressure (Std E. 4.4), skimmer voltage (Std E. 4.3), drying gas temperature (Std E. 4.3), and capillary voltage (Std E. 4.2).

Four factors displayed a consistent statistically significant effect for both CLMN and FBMN workflows with high mean standardized effects: Sample concentration, number of Precursors per cycle, LC duration, and collision energy (Figure 1). These four factors are explored in more detail in Section 2.2.1, Section 2.2.2, Section 2.2.3 and Section 2.2.4. Although fragmentor voltage, sheath gas temperature, skimmer voltage, and nozzle voltage were shown to have a significant effect on molecular network topology, a lower mean standardized effect and inconsistent effect across the three samples show the lesser importance of optimizing these factors.

**Figure 4:**
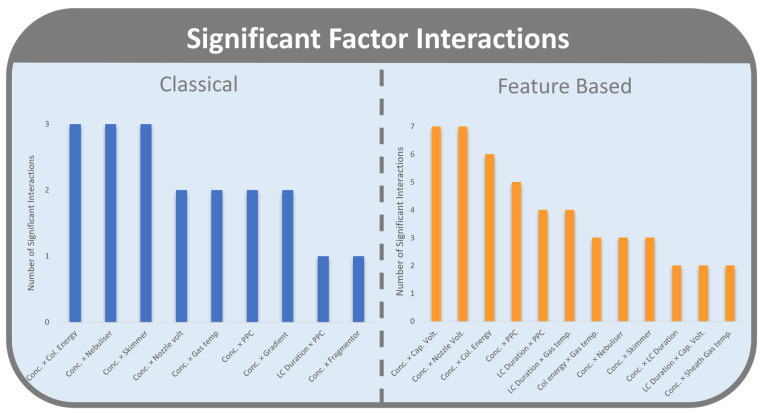
Number of significant factor interactions that affect responses for CLMN (blue, **left**) FBMN (orange, **right**).

Interactions occur when the effect of a factor on a response is dependent on the level of another factor. For CLMN, there were nine significant interactions between factors effecting molecular network responses. These were interactions between concentration and eight other factors (collision energy, nebulizer pressure, skimmer voltage, nozzle voltage, drying gas temperature, precursors per cycle, LC duration, and fragmentor voltage), and between LC duration and precursors per cycle (Figure 4). That concentration was involved in eight of the nine significant factor interactions, further highlights the importance of this factor.

A broader range of interactions was seen in the FBMN workflow, with 12 significant interactions. Nine of these significant interactions were seen between sample concentration and other factors (capillary voltage, nozzle voltage, collision energy, precursors per cycle, nebulizer pressure, skimmer voltage, LC duration, and sheath gas temperature). LC duration had further significant interactions with precursors per cycle, drying gas temperature, and capillary voltage. 

#### 2.2.1. Precursor per Cycle

**Figure 5:**
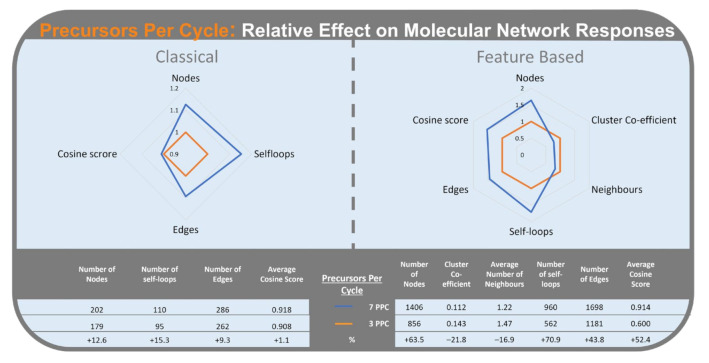
Radar graph of the number of precursors per cycle effect (3 precursors per cycle in orange; 7 precursors per cycle in blue) on the relative change of responses, averaged across the three samples. Classical Molecular Networking (**left**) and Feature-Based Molecular Networking (**right**).

The number of precursors per cycle (PPC) had the third largest Standardized Effect in CLMN and was a significant factor for the four responses we considered for this workflow (Figure 3). Higher PPC (Figure 5) resulted in an increase in self-loop nodes (15.3%), number of nodes (12.6%), number of edges (9.3%), and average cosine score (1.1%).

For FBMN, PPC had the highest Standardized Effect on molecular network topology and significantly affected all responses. Higher PPC increased the number of self-loop nodes (70.9%), number of nodes (63.5%), cosine score (52.4%), and number of edges (43.8%). Higher PPC also reduced the cluster co-efficient (−21.8%) and the average number of neighbours (−16.9%).

PPC consistently affected the number of nodes, number of self-loop nodes, and number of edges. More PPC results in more analytes being chosen for fragmentation per cycle, reducing competition between parent ions. As more analytes are being selected for MS^2^ fragmentation, more nodes appear in the network. Increases in the number of edges with more PPC may result from more minor metabolites being selected for MS^2^ fragmentation; these would have been outcompeted with a lower number of PPC. 

**Figure 6:**
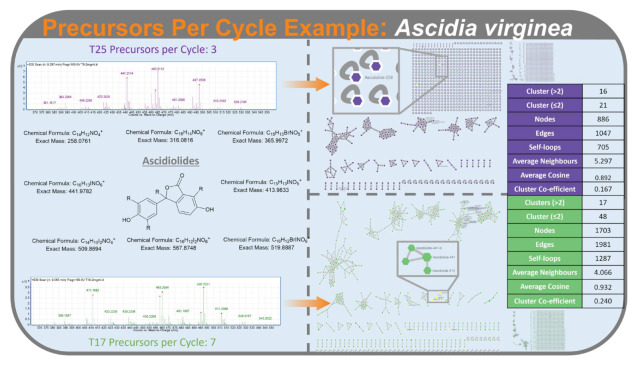
An example of the effects of PPC on molecular networking responses, of a Feature-Based Molecular Network for *Ascidia virginea*. MS spectra with ions selected form MS^2^ fragmentation denoted with a red dot for precursor per cycle of 3 (top, purple, T25) and 7 (bottom, green, T17). Molecular networks produced from these parameters are displayed on the right, with tables displaying molecular network statistics. Eight ascidiolides were annotated with T17 molecular network (7 precursors per cycle) and three ascidiolides were annotated in T25 molecular network (3 precursors per cycle).

In FBMN, higher PPC caused a decline in the number of neighbours and clustering co-efficient. As both values are calculated as an average for the whole network, this reflects the increase in the number of self-loop nodes. The number of clusters with just two nodes increases considerably at the higher level of PPC compared with the lower level. Since two node clusters are awarded a clustering co-efficient of 0, this translates to a decline in average clustering co-efficient for the network. This effect can be seen in the FBMN of *Ascidia virginea* under the condition of T25 (3 precursors per cycle) and T17 (7 precursors per cycle (Figure 6).

#### 2.2.2. Collision Energy

**Figure 7:**
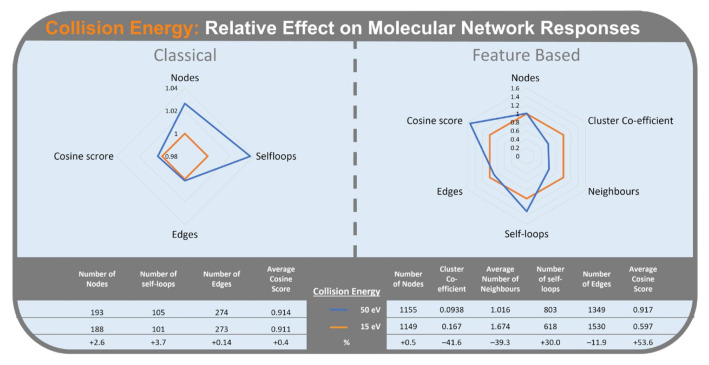
Radar graph of collision energy effect (15 eV in orange) (50 eV in blue) on the relative change of responses. Classical Molecular Networking (**left**) and Feature-Based Molecular Networking (**right**).

In the CLMN workflow, collision energy (Figure 7) had the fourth largest mean Standardized Effect. Increasing collision energy from 15 eV to 50 eV increased number of self-loop nodes (3.7%), number of nodes (2.6%), average cosine score (0.4%), and number of edges (0.14%).

In the FBMN workflow, collision energy had the second highest mean Standardized Effect. Higher collision energy resulted in an increase in average cosine score (53.6%) and number of self-loop nodes (+30%). Higher collision energy also resulted in a decrease in cluster co-efficient (−41.6%), average number of neighbours (−39.3%), and number of edges (−11.9%). Collison energy did not significantly affect the number of nodes in FBMN.

Increasing collision energy generates a higher number of fragments in MS^2^ spectra for a given precursor ion. This provides more points of reference when comparing MS^2^ spectra, leading to the increase in cosine score seen in both CLMN and FBMN. This increase in cosine score, combined with the decrease in number of edges in FBMN, indicates that while fewer similarities between metabolites are detected, the similarities (edges) are being detected more accurately (with higher cosine score). The decrease in cluster co-efficient and average number of neighbours appears to be a result of the combined effect of the increase in self-loop nodes (+30%) and decrease in number of edges (−11.9%). As the edges become more accurate due to more datapoints, matching between nodes is reduced, in turn reducing number of edges, neighbours and cluster size.

**Figure 8:**
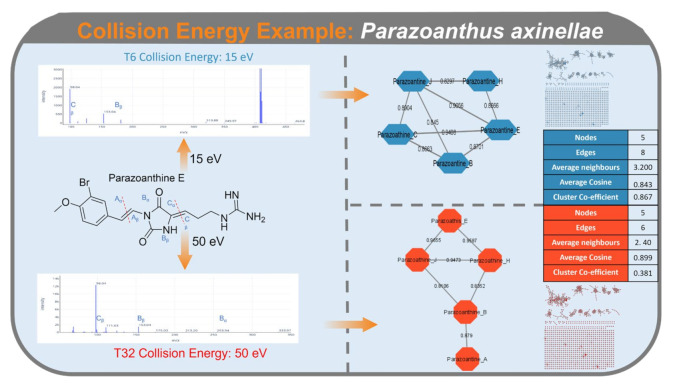
Fragmentation pattern and MS^2^ spectra of parazoanthine E using a collision energy of 15 eV (top, blue, T6) and 50 eV (bottom, red, T32). Parazoanthine clusters with annotated nodes, resulting from a collision energy of 15 eV (top, blue, T6) and 50 eV (bottom, red, T32). Edges are labeled with cosine scores. Tables with cluster statistics under both conditions are displayed on the right.

An example of collision energy effect on molecular network topology can be seen when comparing FBMN of *Parazoanthus axinellae* under parameters set for T6 (collision energy of 15 eV) and T32 (collision energy of 50 eV). The MS^2^ spectra produced with a collision energy of 50 eV resulted in a higher number of fragments produced from parazoanthine E and a parazoanthine cluster with a lower number of edges and higher average cosine scores when compared to the same MS^2^ spectra and cluster which used a collision energy of 15 eV (Figure 8). 

As the effect of collision energy on fragmentation pattern is dependent on the parent ions chemical structure, the increase in the number of fragments observed for parazoanthine E was not seen with the meroterpenoids of the *Halidrys siliquosa,* or the ascidiolides detected in *Ascidia virginea.* This highlights the importance of optimizing the collision energy for the particular type of chemistries within the samples.

#### 2.2.3. Concentration

**Figure 9:**
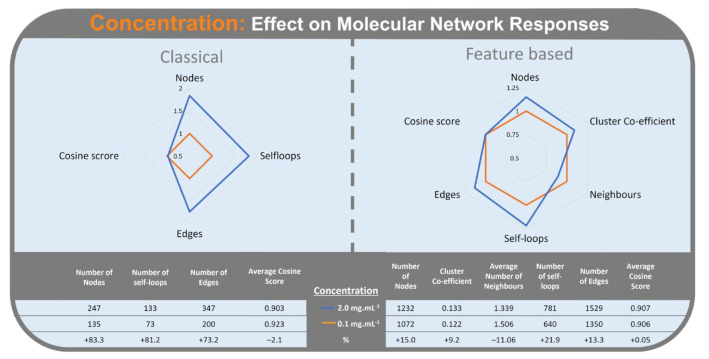
Radar graph of concentration (0.1 mg/mL in orange) (2.0 mg/mL in blue) on the relative change of responses. Classical Molecular Networking (**left**) and Feature-Based Molecular Networking (**right**).

Concentration (Figure 9) had the highest Standardized Effect on CLMN responses. The highest concentration of 2.0 mg/mL increased the number of nodes (83.3%), number of self-loop node (81.2%) and the number of edges (73.2%), when compared with the lowest concentration of 0.1 mg/mL. Average cosine score decreased (−2.1%) with the highest concentration. 

For FBMN, concentration had the third highest Standardized Effect on molecular network responses. Increasing concentration (Figure 9) from 0.1 mg/mL to 2 mg/mL, increased the number of self-loop nodes (21.9%), number of nodes (15.0%), number of edges (13.3%), and cluster co-efficient (9.2%). The average number of neighbours decreased (−11.1%) at the highest concentration. 

As expected, these results indicate that more analytes are detected as concentration increases. This result must be due to the detection of minor metabolites in the sample, which at low concentration are undetected in molecular networking as they do not pass the ion intensity threshold for MS^2^ fragmentation. Increasing the sample concentration increases the ion intensity of these minor metabolites, allowing them to pass the threshold and be represented in the network. Increase in cluster coefficient and average number of edges in the FBMN workflow supports the hypothesis of more minor metabolites being detected with higher concentrations. The increase in self-loop nodes, seen in both CLMN and FBMN, may be a consequence of low intensity ions producing MS^2^ spectra with a higher signal/noise ratio. These high noise spectra are difficult for the molecular networking algorithms to process, and result in an increase in self-loop nodes, as fragment matching is interrupted by increased noise. The variation in the effect of concentration on the number of self-loop nodes in CLMN versus FBMN is likely due to the more advanced data processing used in FBMN. 

**Figure 10:**
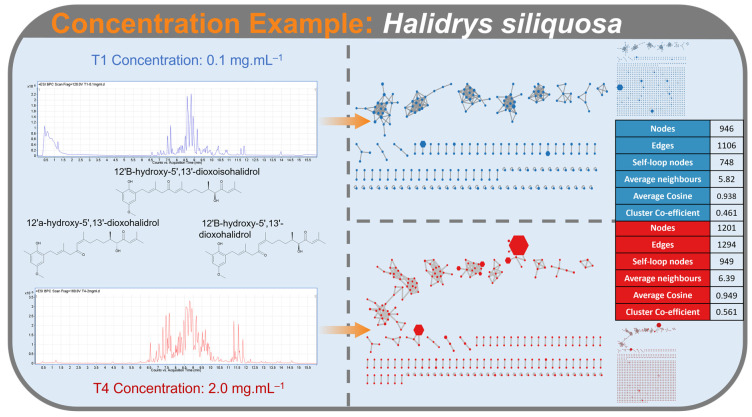
An example comparing the effect of low (0.1 mg/mL; **left**) and high (2.0 mg/mL; **right**) concentrations on Feature-Based Molecular Networking responses of *Halidrys siliquosa*.

This hypothesis is supported when comparing the number of nodes in a low and high concentration molecular network. Comparison of equivalent clusters from the FBMN of the seaweed *Halidrys siliquosa* from run T5 (0.1 mg/mL) and run T17 (2.0 mg/mL), where only concentration differs, shows that at the highest concentration clusters in the network become more populated with low intensity metabolites being represented (Figure 10).

#### 2.2.4. LC Duration 

**Figure 11:**
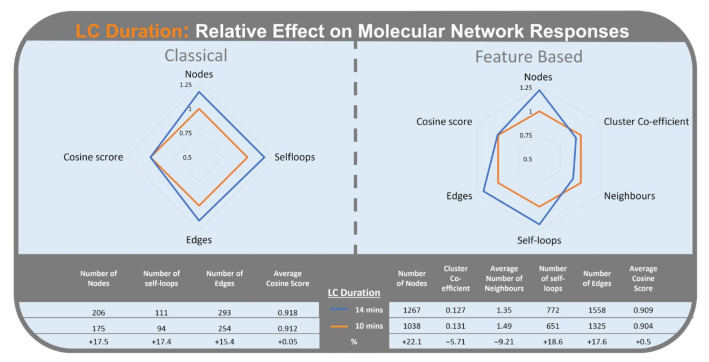
Radar graph of the effect of liquid chromatography duration (10 min in orange) (14 min in blue) on the relative change of responses of Classical Molecular Networking (**left**) and Feature-Based Molecular Networking (**right**).

LC duration had the second highest mean Standardized Effect on CLMN responses (Figure 3). Increased LC duration (Figure 11) resulted in an increase in the number of nodes (17.5%), number of self-loop nodes (17.4%), and number of neighbours (15.4%). LC duration did not have a significant effect on average cosine score (0.05%) in CLMN.

In the FBMN workflow, LC duration had the fourth highest mean Standardized Effect (Std. E. 14.5), with the longer LC duration resulting in an increase of the number of nodes (22.1%), number of self-loop nodes (18.6%), number of edges (17.6%), and average cosine score (0.5%). Average number of neighbours (−9.21%) and cluster co-efficient (−5.7%) decreased with the longer LC duration (Figure 11). 

**Figure 12:**
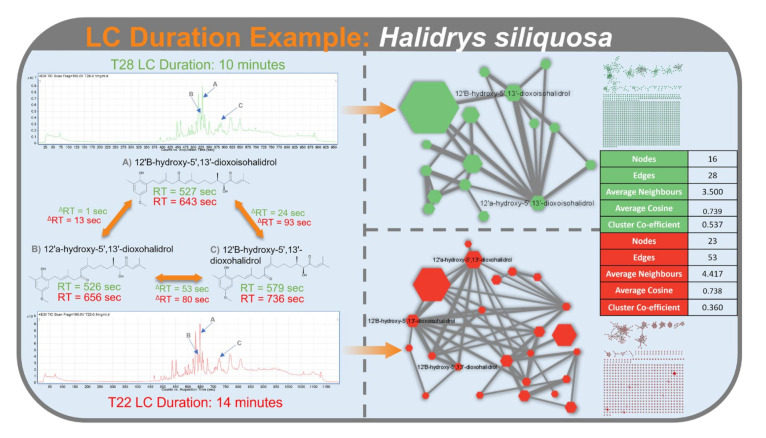
LC-MS^2^ trace of *Halidrys siliquosa* using a LC duration of 10 min (top; green) and 14 min (bottom; red) with the retention times of the meroterpenoids under both conditions displayed. Cluster statistics for the meroterpenoid clusters for both LC-MS^2^ runs are displayed on the right.

An increase in LC duration improves separation between analytes (Figure 12), which reduces the number of analytes entering the mass spectrometer at any one time. As the number of parent ions that can undergo MS^2^ fragmentation per MS scan is limited due to factors including number of precursors per cycle and exclusion time, we hypothesize that greater separation between analytes decreases the competition among parent ions for MS^2^ fragmentation. This reduces competition, results in more analytes undergoing MS^2^ fragmentation, corresponding to the increase observed in the number of nodes and edges in CLMN and FBMN. As the FBMN workflow utilizes retention time of analytes in the feature detection and alignment step, the increase in separation between analytes may benefit the detection of isomers/stereoisomers. This may contribute to the increase in number of nodes and edges. 

#### 2.2.5. Comparison of CLMN and FBMN

According to the results obtained, data acquisition parameters had a larger and more consistent effect on FBMN than CLMN (Figure 3). For FBMN, the significant effects and their Standardized Effect values were consistent across all three samples, whereas for CLMN inconsistencies between the three samples were observed (Table S8). The higher rate of error associated with MSCluster, such as chimeric spectra represented by one node, could have translated into increased error with network statistics and therefore inaccuracies in the fractional factorial analysis. The increased reproducibility of FBMN, due to processing steps such as feature detection and alignment, resulted in more accurate measurements of the effects of parameters. The inclusion of retention times and isotope grouping also allow for more accurate networking, reducing error that would have been present in CLMN. This may be a contributing factor explaining why CLMN results are not as consistent as those for FBMN.

#### 2.2.6. Optimization of Molecular Networking

Overall, the most important parameters to optimize for CLMN are sample concentration and gradient. The use of high concentrations where possible, compatible with the sensitivity of the mass spectrometer used, is recommended. High concentration paired with the longest practical LC duration had a desirable effect on the networks. A high collision energy also appeared beneficial. A higher rate of fragmentation increased cosine scores indicating more accurate edges, improving the analysis of molecular families. For CLMN, the main goal is to visualize the whole chemical space, therefore a high PPC should be used to obtain a high number of nodes representing all the metabolites in the sample. As no in-depth statistical analysis is carried out with CLMN, lower quality data arising from higher PPC do not have as great an impact as they would on FBMN. 

Sample concentration and gradient are also significant factors effecting FBMN topology. As the processing step in FBMN workflow can overcome problems associated with the liquid chromatography part of the analysis (e.g., overlapping peaks), the need to fully optimize these factors is reduced. The use of a higher concentration and longer gradient is still recommended. Increased liquid chromatography separation can improve FBMN’s ability to resolve isomers as the difference in retention times is increased, especially for co-eluting isomers. Collision energy and precursor per cycle had the strongest effect on molecular network topology, and these should be the focus of optimization efforts, in line with the desired responses (e.g., high cosine score vs. low number of self-loop nodes). The use of a response surface Design of Experiment (DoE) could be used to optimize these two factors for the specific chemistry of the samples and aim of constructed molecular network.

As a result of using a screening DoE to generate our fractional factorial design, we could not optimize responses, as only two levels (high/low) were included for each factor (see methods). The ‘response optimizer’ method compares the generated worklist of acquisition parameters to the responses arising from each run and generates optimal data acquisition settings for a given set of responses. For a full optimization, a response surface DoE should be used to optimize the four significant factors (Concentration, Gradient, Collision Energy and Precursor per Cycle) chosen from the screening design.

## 3. Materials and Methods

**Figure 13:**
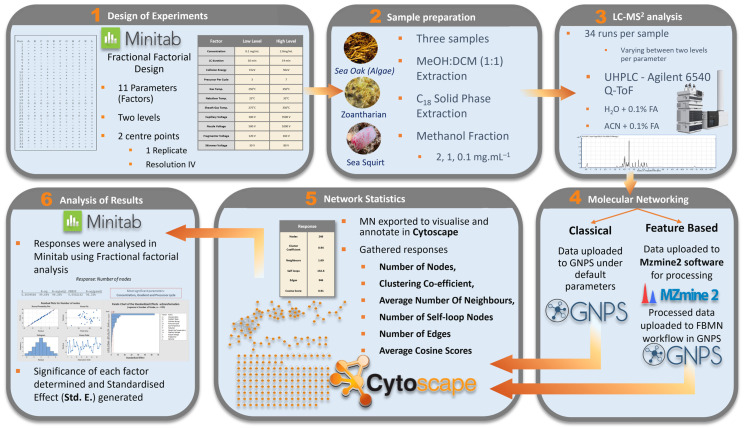
Workflow for evaluating the effect of data acquisition parameters on molecular network topology. Minitab fractional factorial design was used to design an experiment to determine whether a given parameter (factor) significantly affects molecular network topology. Samples were extracted with Methanol: Dichloromethane (1:1) and fractionated on a C_18_ SPE cartridge. A methanolic fraction of each sample was analysed 34 times on a UHPLC-Agilent 6540 q-TOF using unique parameter setting for each analysis. Each parameter had a high and low setting. Molecular networks were generated from untargeted LC-MS^2^ data using both the Classical and Feature-Based workflows offered by the GNPS online platform. Data pre-processing for the FBMN workflow was completed using MZmine 2. Molecular networks from both workflows were vizualised in Cytoscape and responses were analysed in Minitab fractional factorial analysis to determine the Standardised Effect (Std. E.) of each parameter.

The overall workflow of the main experiment is summarized in Figure 13.

### 3.1. Sample Selection and Preparation

Three samples were selected for the main experiment: a sea squirt *Ascidia virginea*, which contained a range of terpene-derived quinone compounds; a zoantharian, *Parazoanthus axinellae*, containing a range of aromatic alkaloids, which were the most polar metabolites of the three samples; and the macroalga, *Halidrys siliquosa*, containing meroterpenoids, the least polar metabolites of the three samples (Figure 1).

For all three samples, 1.0 g of dried biomass was ground using a ball mill and extracted with MeOH/CH_2_Cl_2_ (1:1) under ultrasonification. The extract was fractionated using a 6cc RP-C18 Solid-phase extraction (SPE) cartridge into 4 fractions of decreasing polarity; 100% H_2_O, 1:1 H_2_O: MeOH, 100% MeOH, 1:1 MeOH: DCM using 15 mL of each solvent mixture. The fractions were dried and dissolved in DMSO at a concentration of 10 mg/mL. The 1:1 H_2_O:MeOH and MeOH fractions were combined (1 mL of each) and diluted to obtain the concentrations used in the experimental design.

### 3.2. Experimental Design

Minitab^®^ Statistical Software (Minitab, LLC, Sate College, PA, USA, (2019)) was used to design a resolution IV Fractional Factorial screening experiment, using the Design of Experiment (DoE) function. A single replicate was included, which is sufficient for a screening design. A single replicate design allows the significant factors to be discerned but does not allow for definitive conclusions on factor effects, or for those factors to be optimized. However, since our aim was to discover which factors should be prioritized for optimization, this fractional factorial design (screening DoE) was adequate.

Eleven parameters (factors) were selected in the design: concentration, LC duration, collision energy, precursor per cycle, gas temperature, nebulizer voltage, sheath gas temperature, capillary voltage, nozzle voltage, fragmentor voltage and skimmer voltage, and were each tested at two levels, one low, one high (Table S1). These factors, levels, and their center points were determined based on mass spectrometry knowledge, literature review, GNPS recommendations and Agilent recommendations. The design included two experimental runs with all factors set at the centre point of the two levels, which increases the power of the design. Centre points can be used to determine whether the response surface is linear or curved. The final design had 34 runs per sample (see Table S1) for the settings of each run.

To evaluate the characteristics of the generated molecular networks, six responses were chosen: number of nodes in the network, average number of neighbours, number of self-loop nodes, number of edges, cluster coefficient, and average cosine score. The number of nodes represents the number of metabolites represented by the network. Clustering co-efficient, average number of neighbours and number of edges are indicators of how molecular families are represented in the network. Average cosine scores are determined by the similarity of matches made between nodes in clusters. An increase in self-loop nodes is related to a decrease in clustering co-efficient and a decrease in number of edges, resulting from acquisition parameters that are sub-optimal. Alternatively, lowest intensity ions undergoing fragmentation produce spectra with higher noise ratios, and this noise can be represented by self-loop nodes.

### 3.3. Data Acquisition LC-MS^2^

High Resolution Electrospray Ionization Mass Spectrometry (HRESIMS) data were obtained from a Q-ToF Agilent 6540 in ESI (+) coupled to an Agilent 1290 Infinity II ultra-high performance liquid chromatography system (UHPLC), using a BEH C18 2.1 × 75 mm 1.7 µm column (Acquity, Waters, Milford, CT, USA). Mobile phases of H_2_O (A) + 0.1% FA and CH_3_CN (B) + 0.1% FA were used with a flow set to 0.5 mL/min. An injection volume of 5 µL of sample was used for all LC-MS^2^ experiments. The following gradient was applied: isocratic hold of B at 10% for 2 min followed by increase of B to 100% over a 10 or 14 min (LC duration specified for that run), then an isocratic hold of B at 100% for 4 min and a final decrease of B to 10% over 1 min. A 2-min post-run after each injection for equilibration. Other parameters for a standard LC-MS^2^ experiment were set to low or high values as specified in the fractional factorial experimental design (Table S1).

### 3.4. File Conversion

LC-MS^2^ data were converted from. d (Agilent data format) to. mgf using MS convert, part of the ProteoWizard sofware package [22].

### 3.5. Classical Based Molecular Networking

Molecular networks were created using the online workflow on the GNPS website (http://gnps.ucsd.edu, accessed on 22 February 2022). The data were filtered by removing all MS^2^ fragment ions within +/− 17 Da of the precursor *m*/*z*. MS^2^ spectra were window filtered by choosing only the top six fragment ions in the +/− 50 Da window throughout the spectrum. The precursor ion mass tolerance was set to 2.0 Da and the MS^2^ fragment ion tolerance to 0.5 Da. A network was then created where edges were filtered to have a cosine score above 0.7 and more than six matched peaks. Further, edges between two nodes were kept in the network only if each of the nodes appeared in each other’s respective top 10 most similar nodes. Finally, the maximum size of a molecular family was set to 100, and the lowest scoring edges were removed from molecular families until the molecular family size was below this threshold. The spectra in the network were then searched against GNPS spectral libraries. The library spectra were filtered in the same manner as the input data.

### 3.6. Feature-Based Molecular Networking

LC-MS^2^ data was pre-processed using MzMine2 features including feature detection, chromatogram builder, chromatogram deconvolution and isotopic peaks. Parameters used for processing can be seen in the Supplementary Materials. The processed data were uploaded to GNPS using the FBMN workflow, on the GNPS platform (https://gnps.ucsd.edu, accessed on 22 February 2022). 

### 3.7. Molecular Network Visualization and Network Analyses

Molecular networks were exported to Cytoscape software for visualization. The number of nodes, number of self-loop nodes, clustering-co-efficient and average number of neighbors were generating using the Network Analyser. Networks were treated as undirected. Number of edges and average cosine scores were generated from edge tables.

### 3.8. Design of Experiment Response Analysis

Responses (number of nodes in the network, average number of neighbours, number of self-loop nodes, number of edges, cluster coefficient, and average cosine score) were returned to Minitab for each sample, resulting in 36 response models (6 responses × 3 samples × 2 workflows). Two terms, all main effects (factor effects on responses e.g., concentration effect on cosine score) and 2-way interactions (two factors interacting to effect a response) were selected as model terms for analyzing the factorial design, with no covariates. Two-sided confidence level for all intervals was set to 95%, to estimate the higher and lower values of the mean response. DoE response analysis generated Standardized Effects (Std. E.) for each factor and 2-way interaction effect on a response. Standardized effects incorporate standard deviations of observations and thus allow for the evaluation/comparison of the size of various factor effects that have different units on responses. 

DoE response analysis in Minitab generates multiple outputs for analyzing the effect of each factor/two-way interaction as well as the quality of each response model. Normal plots and half normal plots of standardized effects, and Pareto charts were used to identify and compare the relative magnitude of a factor’s effect on a given response, as well as the statistical significance. A model summary was also generated for each response model, containing S, predicted R^2^ (R^2^ Pred.) and *p*-values which were used to determine the quality of the model. S is a measure, in terms of standard deviations, of how the data values differ from the fitted values and indicates how well the model describes the response. Lower S values indicate a model that better describes the response. Predicted R^2^ is a measure of how well the model can predict a response; higher R^2^ Pred. indicates better predicative ability. The *p*-value is the probability that the null hypothesis (i.e., factor has no effect on responses) is true. Residual plots were generated to detect issues with regression. S values in combination with residual plots were analyzed to identify and excluded any model with poor fitting and/or biased data from response analysis. Models with poor fit (*p*-value > 0.05 or R^2^ pred. < 20) were not included in the response analysis. 

### 3.9. Visualization of Molecular Networking 

Results from DoE response analysis were exported to Microsoft Excel. Factor effect on responses was averaged across the three samples for the CLMN and FBMN workflows. Bar charts were generated to summarize the Standardized Effect of each factor for both workflows. Four- and six-dimensional radar maps were used to illustrate PPC, collision energy, concentration, and LC durations effect on CLMN and FBMN responses respectively.

## 4. Conclusions

When applied to natural products, molecular networking is an incredibly versatile tool, that can be used to help answer a variety of questions. What makes a “good” molecular network depends on the purpose of the molecular network, and the most appropriate workflow, FBMN or CLMN, depends on the question. For a quick analysis of chemodiversity and dereplication, for example, to help prioritize samples for in-depth chemical analysis, CLMN would be preferable, due to the time efficient analysis of the workflow. For in depth analysis, such as metabolomic analyses or samples with known isomers, FBMN is preferable due to its ability to quantify metabolites, resolve isomers/stereoisomers and its increased reproducibility. FBMN could also be preferable when matching data against databases for dereplication as it holds more information allowing for more accurate comparison. 

Once the appropriate workflow is selected, the next step is to determine the optimal responses. To determine whether there are analogues of a known bioactive compound in a sample, high number of nodes, edges, and cluster coefficient should be the responses selected for optimization. If the aim were to compare samples, where statistical accuracy and reproducibility are of high importance, a high cosine score and low number of self-loop nodes should be optimized. 

Preliminary studies were necessary to recognize the need for optimization of molecular networking. The use of statistical tools and software, such as Minitab’s screening DoE feature, is a time-saving and effective way to determine the significance of multiple factors and their interactions. 

Mass spectrometry data acquisition parameters have a significant effect on the network topology and interpretation, with the most significant parameters shown to be concentration, LC duration, collision energy and number of precursors per cycle. When correctly used and interpreted, molecular networking can substantially speed up the dereplication of samples and provides a visual representation of sample components.

## Figures and Tables

**Figure 2:**
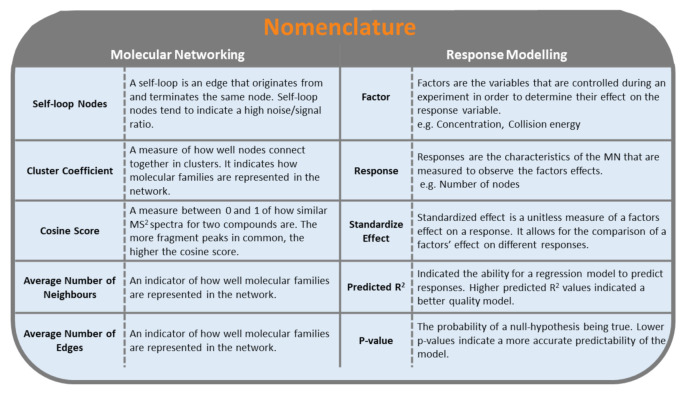
Nomenclature used for molecular networking (**left**) and DoE response modelling (**right**).

## Data Availability

The data presented in this study are openly available in GNPS-MassIVE Datasets at doi:10.25345/C5FN10W04.

## Supplementary Materials

The following are available online at https://www.mdpi.com/article/10.3390/metabo12030245/s1, Figures S1–S3: LC-MS^2^ chromatograms of samples; Figures S4–S39: DoE reports for three samples and two workflows; Table S1: Data acquisition parameters; Tables S2–S7: Molecular networking responses; Table S8: Statistical analysis of response model.
