# Supplementary material for: Optimization of LC-MS2 Data Acquisition Parameters for Molecular Networking Applied to Marine Natural Products
Source: Metabolites. 2022 Mar 14;12(3):245. doi: 10.3390/metabo12030245 (PMC8953742; doi:10.3390/metabo12030245)
Supplement: Supplementary file 1 [file metabolites-12-00245-s001.zip › metabolites-1616265-supplementary.pdf]

Optimization of LC-MS^2 Data Acquisition Parameters for Molecular Networking applied to Marine Natural Products

Sam Afoullouss [1,2], Agata Balsam[1], A. Louise Allcock[2] and Olivier P. Thomas [1*]

[1] School of Biological and Chemical Sciences and Ryan Institute, National University of Ireland Galway (NUI Galway), University Road, H91TK33 Galway, Ireland; s.afoullouss1@nuigalway.ie (SA); A.Balsam1@nuigalway.ie (AB); olivier.thomas@nuigalway.ie (O.T)
[2] School of Natural Sciences and Ryan Institute, National University of Ireland Galway (NUI Galway), University Road, H91TK33 Galway, Ireland; louise.allcock@nuigalway.ie (ALA)
* Correspondence: Olivier.thomas@nuigalway.ie

Figure S1: UPLC-ESI-MS^2 profiles of Ascidia virginea run on a C_{18} column 5
Figure S2: LC-MS^2 profiles of Parazoanthus axinellae run on a C_{18} column. 5
Figure S3: LC-MS^2 profiles of Halidrys siliquosa run on a C_{18} column. 5
Table S1: Table showing the LC-MS^2 parameters (factors) chosen for molecular networking optimization and the levels used for each factor. The order of experiments (right) shows the levels for each factor per run, with + representing the higher level, - representing the lower level and 0 representing the midpoint. 6
Table S2: Responses of molecular networks for 34 experimental conditions on Classical Molecular Networking workflow on sample *Ascidia virginea*. 7
Table S3: Responses of molecular networks for 34 experimental conditions on Classical Molecular Networking workflow on sample *Parazoanthus axinellae*. 8
Table S4: Responses of molecular networks for 34 experimental conditions on Classical Molecular Networking workflow on sample *Halidrys siliquosa*. 9
Table S5: Responses of molecular networks for 34 experimental conditions on Feature-Based Molecular Networking workflow on sample *Ascidia virginea* 10
Table S6: Responses of molecular networks for 34 experimental conditions on Feature-Based Molecular Networking workflow on sample *Parazoanthus axinellae*. 11
Table S7: Responses of molecular networks for 34 experimental conditions on Feature-Based Molecular Networking workflow on sample *Halidrys siliquosa*. 12
Table S8: R^2, R^2 (predicted), R^2 (adjusted), PRESS, S and P statistical values of response models for three samples and two workflows. Models included in summary analysis are indicated with an asterisk. Models highlighted in red were excluded from final analysis because non-significant (R^2<20). 13
Figure S4: DoE reports for number of node model in Classical Molecular Networking of *Ascidia virginea*, normal plot of standardized effects (top left), half normal plot of standardized effects (top right), pareto chart of standardized effects (bottom left), and residual plots (bottom right)…………..14
Figure S5: DoE reports for cluster co-efficient model in Classical Molecular Networking of A*scidia virginea,* normal plot of standardized effects (top left), half normal plot of standardized effects (top right), pareto chart of standardized effects (bottom left), and residual plots (bottom right). 15
Figure S6: DoE reports for average number of neighbours model in Classical Molecular Networking of A*scidia virginea,* normal plot of standardized effects (top left), half normal plot of standardized effects (top right), pareto chart of standardized effects (bottom left), and residual plots (bottom right). 16
Figure S7: DoE reports for number of self-loop nodes model in Classical Molecular Networking of *Ascidia virginea,* normal plot of standardized effects (top left), half normal plot of standardized effects (top right), pareto chart of standardized effects (bottom left), and residual plots (bottom right). 17

Figure S8: DoE reports for number of edges model in Classical Molecular Networking of *Ascidia virginea*, normal plot of standardized effects (top left), half normal plot of standardized effects (top right), pareto chart of standardized effects (bottom left), and residual plots (bottom right). 18
Figure S9: DoE reports for average cosine score model in Classical Molecular Networking of *Ascidia virginea*, normal plot of standardized effects (top left), half normal plot of standardized effects (top right), pareto chart of standardized effects (bottom left), and residual plots (bottom right). 19
Figure S10: DoE reports for number of nodes model in Classical Molecular Networking of *Parazoanthus axinellae*, normal plot of standardized effects (top left), half normal plot of standardized effects (top right), pareto chart of standardized effects (bottom left), and residual plots (bottom right). 20
Figure S11: DoE reports for cluster co-efficient model in Classical Molecular Networking of *Parazoanthus axinellae*, normal plot of standardized effects (top left), half normal plot of standardized effects (top right), pareto chart of standardized effects (bottom left), and residual plots (bottom right). 21
Figure S12: DoE reports for average number of neighbours model in Classical Molecular Networking of *Parazoanthus axinellae*, normal plot of standardized effects (top left), half normal plot of standardized effects (top right), pareto chart of standardized effects (bottom left), and residual plots (bottom right). 22
Figure S13: DoE reports for number of self-loop nodes model in Classical Molecular Networking of *Parazoanthus axinellae*, normal plot of standardized effects (top left), half normal plot of standardized effects (top right), pareto chart of standardized effects (bottom left), and residual plots (bottom right). 23
Figure S14: DoE reports for number of edges model in Classical Molecular Networking of *Parazoanthus axinellae*, normal plot of standardized effects (top left), half normal plot of standardized effects (top right), pareto chart of standardized effects (bottom left), and residual plots (bottom right). 24
Figure S15: DoE reports for average cosine score model in Classical Molecular Networking of *Parazoanthus axinellae*, normal plot of standardized effects (top left), half normal plot of standardized effects (top right), pareto chart of standardized effects (bottom left), and residual plots (bottom right). 25
Figure S16: DoE reports for number of nodes model in Classical Molecular Networking of *Halidrys siliquosa*, normal plot of standardized effects (top left), half normal plot of standardized effects (top right), pareto chart of standardized effects (bottom left), and residual plots (bottom right). 26
Figure S17: DoE reports for cluster co-efficient model in Classical Molecular Networking of *Halidrys siliquosa*, normal plot of standardized effects (top left), half normal plot of standardized effects (top right), pareto chart of standardized effects (bottom left), and residual plots (bottom right). 27
Figure S18: DoE reports for average number of neighbours model in Classical Molecular Networking of *Halidrys siliquosa,* normal plot of standardized effects (top left), half normal plot of standardized effects (top right), pareto chart of standardized effects (bottom left), and residual plots (bottom right). 28
Figure S19: DoE reports for number of self-loop nodes model in Classical Molecular Networking of Halidrys siliquosa, normal plot of standardized effects (top left), half normal plot of standardized effects (top right), pareto chart of standardized effects (bottom left), and residual plots (bottom right). 29
Figure S20: DoE reports for number of nodes model in Classical Molecular Networking of *Halidrys siliquosa,* normal plot of standardized effects (top left), half normal plot of standardized effects (top right), pareto chart of standardized effects (bottom left), and residual plots (bottom right). 30
Figure S21: DoE reports for average cosine model in Classical Molecular Networking of *Halidrys siliquosa,* normal plot of standardized effects (top left), half normal plot of standardized effects (top right), pareto chart of standardized effects (bottom left), and residual plots (bottom right). 31
Figure S22: DoE reports for number of node model in Feature-Based Molecular Networking of *Ascidia virginea,* normal plot of standardized effects (top left), half normal plot of standardized effects (top right), pareto chart of standardized effects (bottom left), and residual plots (bottom right). 32

Figure S23: DoE reports for cluster co-efficient model in Feature-Based Molecular Networking of *Ascidia virginea*, normal plot of standardized effects (top left), half normal plot of standardized effects (top right), pareto chart of standardized effects (bottom left), and residual plots (bottom right). 33
Figure S24: DoE reports for average number of neighbours model in Feature-Based Molecular Networking of *Ascidia virginea,* normal plot of standardized effects (top left), half normal plot of standardized effects (top right), pareto chart of standardized effects (bottom left), and residual plots (bottom right). 34
Figure S25: DoE reports for number of self-loop nodes model in Feature-Based Molecular Networking of *Ascidia virginea,* normal plot of standardized effects (top left), half normal plot of standardized effects (top right), pareto chart of standardized effects (bottom left), and residual plots (bottom right).......... 35
Figure S26: DoE reports for number of edges model in Feature-Based Molecular Networking of *Ascidia virginea,* normal plot of standardized effects (top left), half normal plot of standardized effects (top right), pareto chart of standardized effects (bottom left), and residual plots (bottom right). 36
Figure S27: DoE reports for average cosine score model in Feature-Based Molecular Networking of *Ascidia virginea* normal plot of standardized effects (top left), half normal plot of standardized effects (top right), pareto chart of standardized effects (bottom left), and residual plots (bottom right).......... 37
Figure S28: DoE reports for number of node model in Feature-Based Molecular Networking of *Parazoanthus axinellae*, normal plot of standardized effects (top left), half normal plot of standardized effects (top right), pareto chart of standardized effects (bottom left), and residual plots (bottom right). 38
Figure S29: DoE reports for cluster co-efficient model in Feature-Based Molecular Networking of *Parazoanthus axinellae,* normal plot of standardized effects (top left), half normal plot of standardized effects (top right), pareto chart of standardized effects (bottom left), and residual plots (bottom right). 39
Figure S30: DoE reports for average number of neighbours model in Feature-Based Molecular Networking of *Parazoanthus axinellae*, normal plot of standardized effects (top left), half normal plot of standardized effects (top right), pareto chart of standardized effects (bottom left), and residual plots (bottom right). 40
Figure S31: DoE reports for number of self-loop nodes model in Feature-Based Molecular Networking of *Parazoanthus axinellae*, normal plot of standardized effects (top left), half normal plot of standardized effects (top right), pareto chart of standardized effects (bottom left), and residual plots (bottom right). 41
Figure S32: DoE reports for number of edges model in Feature-Based Molecular Networking of *Parazoanthus axinellae*, normal plot of standardized effects (top left), half normal plot of standardized effects (top right), pareto chart of standardized effects (bottom left), and residual plots (bottom right). 42
Figure S33: DoE reports for average cosine score model in Feature-Based Molecular Networking of *Parazoanthus axinellae,* normal plot of standardized effects (top left), half normal plot of standardized effects (top right), pareto chart of standardized effects (bottom left), and residual plots (bottom right). 43
Figure S34: DoE reports for number of nodes model in Feature-Based Molecular Networking of *Halidrys siliquosa*, normal plot of standardized effects (top left), half normal plot of standardized effects (top right), pareto chart of standardized effects (bottom left), and residual plots (bottom right). 44
Figure S35: DoE reports for cluster co-efficient model in Feature-Based Molecular Networking of *Halidrys siliquosa*, normal plot of standardized effects (top left), half normal plot of standardized effects (top right), pareto chart of standardized effects (bottom left), and residual plots (bottom right).......... 45
Figure S36: : DoE reports for cluster co-efficient model in Feature-Based Molecular Networking of *Halidrys siliquosa,* normal plot of standardized effects (top left), half normal plot of standardized effects (top right), pareto chart of standardized effects (bottom left), and residual plots (bottom right).......... 46
Figure S37: DoE reports for number of self-loop nodes model in Feature-Based Molecular Networking of *Halidrys siliquosa,* normal plot of standardized effects (top left), half normal plot of standardized

effects (top right), pareto chart of standardized effects (bottom left), and residual plots (bottom right). 47
Figure S38: DoE reports for number of edges model in Feature-Based Molecular Networking of *Halidrys siliquosa,* normal plot of standardized effects (top left), half normal plot of standardized effects (top right), pareto chart of standardized effects (bottom left), and residual plots (bottom right). 48
Figure S39: DoE reports for average cosine score model in Feature-Based Molecular Networking of *Halidrys siliquosa,* normal plot of standardized effects (top left), half normal plot of standardized effects (top right), pareto chart of standardized effects (bottom left), and residual plots (bottom right). 49

LC-MS profiles

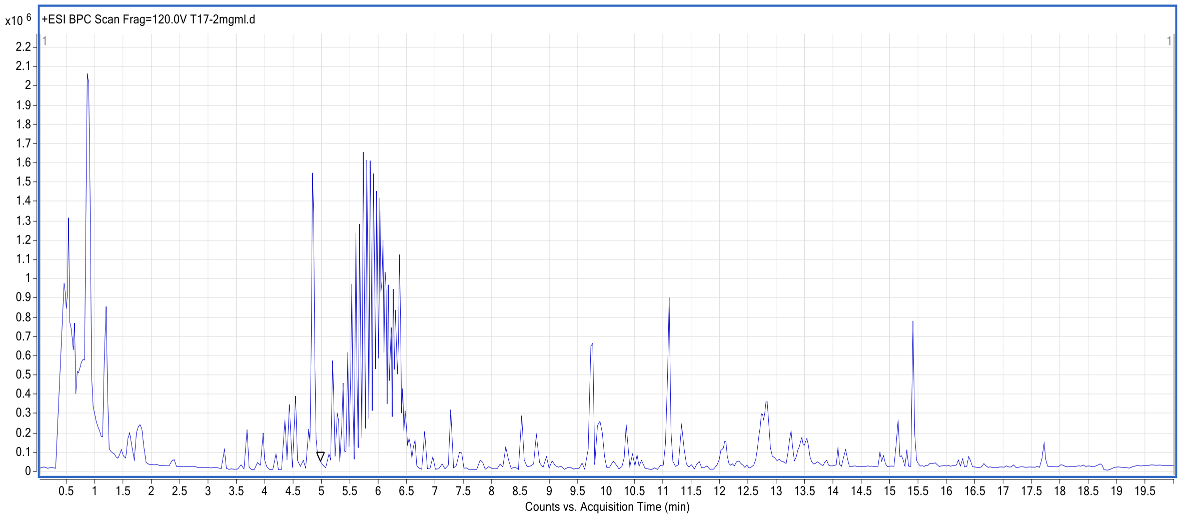

Figure S1: UPLC-ESI-MS² profiles of *Ascidia virginea* run on a C18 column

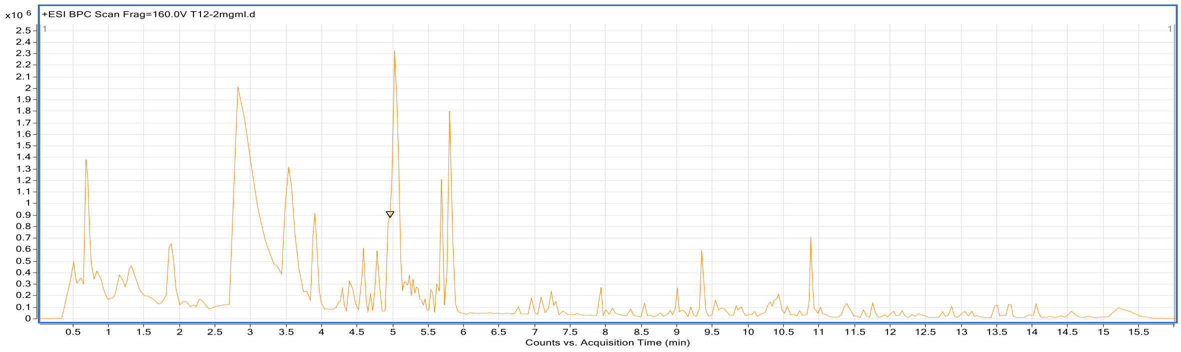

Figure S2: LC-MS² profiles of *Parazoanthus axinellae* run on a C18 column.

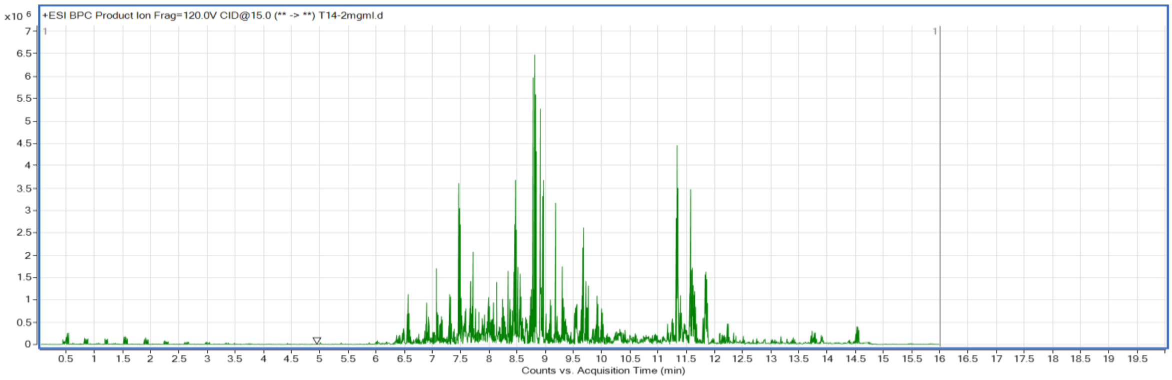

Figure S3: LC-MS² profiles of *Halidrys siliquosa* run on a C18 column.

Design of Experiment (DOE)

	Factor	Low Level (-)	Centre Point (0)	High Level (+)
**A**	**Concentration**	0.1 mg/mL	1 mg/ mL	2.0mg/mL
**B**	**LC duration**	10 min	12 min	14 min
**C**	**Collision Energy**	15 eV	30 eV	50 eV
**D**	**Precursor Per Cycle**	3	5	7
**E**	**Gas Temp.**	250°C	300°C	350°C
**F**	**Nebuliser Pressure**	25 PSI	30 PSI	35 PSI
**G**	**Sheath Gas Temp.**	275°C	310°C	350°C
**H**	**Capillary Voltage**	300 V	1750 V	3500 V
**J**	**Nozzle Voltage**	500 V	750 V	1000 V
**K**	**Fragmentor Voltage**	120 V	140 V	160 V
**L**	**Skimmer Voltage**	30 V	55 V	80 V

DOE

Run	A	B	C	D	E	F	G	H	J	K	L
1	-	-	+	+	+	+	-	+	-	-	-
2	-	+	+	+	+	-	+	+	-	-	+
3	-	+	-	-	-	+	+	-	-	-	+
4	+	-	+	+	+	-	-	+	+	+	-
5	-	+	-	+	-	+	-	+	+	+	-
6	+	+	-	-	-	-	+	-	+	+	+
7	+	-	-	-	-	+	-	-	+	+	-
8	-	-	+	-	-	+	+	+	+	-	-
9	+	-	+	+	-	-	-	-	+	-	+
10	-	-	-	+	+	-	+	-	+	-	-
11	-	+	+	+	-	-	+	-	-	+	-
12	+	-	-	+	+	+	+	-	-	+	-
13	-	-	-	-	-	-	-	-	-	-	-
14	+	-	-	-	+	+	-	+	+	-	+
15	+	-	-	+	-	+	+	+	-	-	+
16	+	-	+	-	+	-	+	-	-	-	+
17	+	+	+	+	-	+	+	-	+	-	-
18	+	+	-	+	+	-	-	-	-	+	+
19	+	-	+	-	-	-	+	+	-	+	-
20	0	0	0	0	0	0	0	0	0	0	0
21	+	+	-	+	-	-	-	+	-	-	-
22	-	+	-	-	+	+	+	+	-	+	-
23	-	+	-	+	+	+	-	-	+	-	+
24	0	0	0	0	0	0	0	0	0	0	0
25	+	+	+	-	-	+	-	+	-	+	+
26	-	-	+	-	+	+	+	-	+	+	+
27	-	-	+	+	-	+	-	-	-	+	+
28	-	-	-	-	+	-	-	+	-	+	+
29	+	+	+	+	+	+	+	+	+	+	+
30	-	+	+	-	+	-	-	-	+	+	-
31	+	+	-	-	+	-	+	+	+	-	-
32	+	+	+	-	+	+	-	-	-	-	-
33	-	+	+	-	-	-	-	+	+	-	+
34	-	-	-	+	-	-	+	+	+	+	+

Table S1: Table showing the LC-MS² parameters (factors) chosen for molecular networking optimization and the levels used for each factor. The order of experiments (right) shows the levels for each factor per run, with + representing the higher level, - representing the lower level and 0 representing the midpoint.

Network statistics (Cytoscape)

Run	# Nodes	Cluster co-efficient	Average # neighbours	# self-loop nodes	#Edges	Average Cosine score
T1	131	0.182	2.458	64	225	0.8828
T2	181	0.215	1.945	93	269	0.9136
T3	155	0.163	2.155	73	240	0.8999
T4	263	0.224	2.167	138	423	0.90897
T5	151	0.204	1.96	66	214	0.8963
T6	274	0.221	2.438	139	473	0.8736
T7	222	0.249	2.324	111	369	0.8717
T8	141	0.217	2.312	70	233	0.9072
T9	282	0.17	2.113	142	440	0.9011
T10	147	0.193	2.054	61	212	0.9023
T11	173	0.186	1.919	99	265	0.9342
T12	268	0.256	2.276	141	446	0.8721
T13	122	0.154	2.049	60	185	0.9075
T14	236	0.233	2.602	108	415	0.8619
T15	275	0.21	2.073	134	419	0.8739
T16	270	0.181	2.111	146	431	0.9241
T17	330	0.167	2.139	184	537	0.91599
T18	311	0.209	2.238	146	494	0.863
T19	221	0.207	2.335	113	371	0.9304
T20	289	0.2	2.048	131	427	0.9079
T21	238	0.201	2	128	366	0.8746
T22	135	0.223	2.222	65	215	0.9114
T23	148	0.15	1.865	79	217	0.9099
T24	295	0.274	2.285	137	474	0.8995
T25	303	0.267	2.224	137	454	0.908
T26	138	0.271	2.522	68	242	0.9098
T27	148	0.175	2.108	77	233	0.8996
T28	128	0.144	1.7344	67	178	0.9063
T29	363	0.161	1.906	198	544	0.9233
T30	152	0.239	2.434	67	252	0.8968
T31	271	0.226	2.458	120	453	0.8645
T32	261	0.225	2.521	118	447	0.9007
T33	153	0.265	2.627	68	269	0.8879
T34	158	0.184	1.975	78	234	0.9034

Table S2: Responses of molecular networks for 34 experimental conditions on Classical Molecular Networking workflow on sample *Ascidia virginea.*

Run	# Nodes	Cluster co-efficient	Average # neighbours	# self-loop nodes	#Edges	Average Cosine score
T1	116	0.083	1.138	77	143	0.941
T2	134	0.148	1.343	87	177	0.947
T3	150	0.188	2.12	87	146	0.924
T4	211	0.207	1.583	118	285	0.906
T5	114	0.198	2.702	56	210	0.924
T6	278	0.257	1.763	146	391	0.887
T7	233	0.189	1.511	129	305	0.907
T8	137	0.134	1.08	83	157	0.921
T9	260	0.181	1.777	141	372	0.898
T10	124	0.215	2.177	59	194	0.91
T11	132	0.161	1.439	84	179	0.933
T12	245	0.142	1.331	132	295	0.905
T13	124	0.173	2.1777	67	202	0.923
T14	245	0.134	1.518	133	319	0.9
T15	241	0.151	1.502	128	309	0.909
T16	252	0.258	2.079	116	378	0.891
T17	277	0.126	1.3	170	350	0.921
T18	290	0.154	1.297	161	349	0.902
T19	218	0.219	1.899	103	310	0.902
T20	254	0.305	2.307	108	401	0.891
T21	206	0.142	1.194	125	248	0.919
T22	133	0.19	2.165	75	219	0.914
T23	147	0.175	2.054	75	226	0.915
T24	243	0.215	1.877	122	350	0.908
T25	283	0.198	1.972	149	428	0.905
T26	126	0.253	1.825	63	178	0.909
T27	97	0.155	1.278	51	113	0.927
T28	126	0.185	1.667	73	178	0.93
T29	326	0.191	1.687	187	462	0.907
T30	120	0.157	1.383	66	149	0.909
T31	262	0.214	1.794	131	366	0.886
T32	258	0.228	2.078	140	408	0.899
T33	119	0.188	1.227	68	141	0.914
T34	115	0.171	2.539	56	202	0.919

Table S3: Responses of molecular networks for 34 experimental conditions on Classical Molecular Networking workflow on sample *Parazoanthus axinellae*.

Run	# Nodes	Cluster co-efficient	Average # neighbours	# self-loop nodes	#Edges	Average Cosine score
T1	122	0.156	1.525	75	168	0.924
T2	144	0.155	1.514	77	186	0.937
T3	161	0.163	1.963	89	247	0.921
T4	200	0.151	1.18	112	230	0.892
T5	167	0.178	2.12	79	256	0.887
T6	215	0.134	1.274	119	256	0.901
T7	155	0.092	1.019	86	165	0.904
T8	113	0.111	1.628	61	153	0.925
T9	203	0.121	1.113	116	229	0.899
T10	144	0.24	2.431	68	243	0.902
T11	155	0.133	1.342	92	196	0.945
T12	209	0.091	1.244	140	270	0.922
T13	21	0.186	2.281	60	198	0.896
T14	158	0.028	0.443	111	146	0.953
T15	213	0.117	1.418	122	273	0.901
T16	181	0.205	1.514	85	222	0.878
T17	282	0.126	1.333	158	346	0.901
T18	269	0.125	1.242	163	330	0.91
T19	158	0.107	0.975	99	176	0.917
T20	237	0.14	1.207	142	285	0.915
T21	245	0.149	1.567	130	322	0.888
T22	152	0.153	1.934	92	239	0.92
T23	172	0.163	1.733	89	238	0.909
T24	236	0.156	1.178	154	293	0.923
T25	199	0.085	0.945	116	210	0.907
T26	115	0.094	1.426	67	149	0.93
T27	133	0.147	1.564	80	184	0.917
T28	112	0.149	1.339	62	137	0.904
T29	276	0.153	1.29	161	339	0.903
T30	114	0.098	1.14	75	140	0.934
T31	209	0.14	1.196	137	262	0.929
T32	195	0.14	1.221	100	219	0.888
T33	102	0.147	1.373	61	131	0.925
T34	156	0.181	2.192	83	254	0.905

Table S4: Responses of molecular networks for 34 experimental conditions on Classical Molecular Networking workflow on sample *Halidrys siliquosa*.

Run	# Nodes	Cluster co-efficient	Average # neighbours	# self-loop nodes	#Edges	Average Cosine score
T1	1131	0.095	1.149	816	1466	0.917
T2	1443	0.081	0.99	1037	1751	0.929
T3	925	0.177	2.017	504	1437	0.894
T4	1373	0.067	0.721	1086	1581	0.944
T5	1356	0.109	1.572	803	1869	0.899
T6	1064	0.149	1.485	618	1408	0.901
T7	851	0.174	1.556	469	1131	0.895
T8	748	0.14	1.463	497	1044	0.899
T9	1391	0.092	0.95	1083	1744	0.925
T10	1141	0.15	1.939	615	1721	0.892
T11	1357	0.067	0.856	966	1547	0.932
T12	1428	0.118	1.195	895	1748	0.915
T13	763	0.195	2.168	381	1208	0.894
T14	892	0.199	1.774	381	1268	0.887
T15	1385	0.124	1.225	909	1757	0.919
T16	860	0.12	1.209	604	1124	0.914
T17	1703	0.07	0.794	1305	1981	0.932
T18	1704	0.121	1.192	1088	2104	0.913
T19	883	0.085	0.879	656	1044	0.94
T20	1271	0.142	1.405	826	1719	0.898
T21	1527	0.093	0.968	1040	1779	0.932
T22	898	0.164	2.011	471	1374	0.89
T23	1507	0.112	1.664	845	2099	0.897
T24	1283	0.135	1.32	844	1691	0.903
T25	1024	0.093	0.908	760	1225	0.921
T26	760	0.11	1.232	527	995	0.906
T27	1212	0.1	1.101	860	1527	0.918
T28	786	0.167	2.041	410	1212	0.892
T29	1738	0.067	0.746	1331	1979	0.934
T30	947	0.084	1.026	690	1176	0.916
T31	1087	0.149	1.529	617	1448	0.9
T32	1055	0.088	0.963	772	1280	0.919
T33	948	0.107	1.399	628	1291	0.905
T34	1128	0.134	1.711	677	1642	0.903

Table S5: Responses of molecular networks for 34 experimental conditions on Feature-Based Molecular Networking workflow on sample *Ascidia virginea*

Run	# Nodes	Cluster co-efficient	Average # neighbours	# self-loop nodes	#Edges	Average Cosine score
T1	947	0.069	0.788	729	1102	0.935
T2	1163	0.068	0.717	883	1300	0.946
T3	807	0.131	1.492	521	1123	0.917
T4	1201	0.066	0.664	944	1343	0.95
T5	1148	0.12	1.287	672	1411	0.906
T6	943	0.167	1.561	565	1301	0.9
T7	735	0.173	1.616	432	1026	0.894
T8	646	0.115	1.031	474	807	0.919
T9	1281	0.102	0.831	979	1511	0.927
T10	1007	0.13	1.472	622	1363	0.911
T11	1145	0.053	0.624	874	1231	0.948
T12	1222	0.116	1.275	768	1547	0.912
T13	656	0.168	1.796	419	1008	0.899
T14	786	0.18	1.616	421	1056	0.895
T15	1237	0.136	1.384	804	1660	0.912
T16	770	0.121	1.158	540	986	0.907
T17	1457	0.08	0.729	1130	1661	0.933
T18	517	0.495	2.381	288	2094	0.936
T19	772	0.117	1.096	554	977	0.914
T20	1075	0.128	1.146	746	1362	0.914
T21	1350	0.131	1.25	894	1738	0.911
T22	752	0.154	1.739	448	1102	0.894
T23	1230	0.139	1.577	708	1678	0.895
T24	1043	0.119	1.141	707	1302	0.909
T25	886	0.109	1.005	676	1121	0.915
T26	682	0.102	0.933	520	838	0.92
T27	1003	0.067	0.704	782	1135	0.938
T28	665	0.172	0.704	383	969	0.889
T29	1470	0.08	0.837	1123	1738	0.925
T30	762	0.085	0.801	586	891	0.925
T31	937	0.17	1.639	554	1322	0.9
T32	937	0.107	0.95	705	1150	0.921
T33	767	0.101	0.92	588	942	0.918
T34	974	0.138	1.509	558	1293	0.901

Table S6: Responses of molecular networks for 34 experimental conditions on Feature-Based Molecular Networking workflow on sample *Parazoanthus axinellae*.

Run	# Nodes	Cluster co-efficient	Average # neighbours	# self-loop nodes	#Edges	Average Cosine score
T1	1210	0.09	1.069	872	1519	0.926
T2	1465	0.073	0.995	1045	1774	0.929
T3	951	0.181	2.038	484	1453	0.892
T4	1284	0.084	0.984	915	1547	0.918
T5	1519	0.111	1.531	837	2000	0.897
T6	1118	0.195	2.159	528	1735	0.877
T7	849	0.2	2.174	414	1337	0.868
T8	756	0.137	1.534	478	1058	0.898
T9	1380	0.093	1.293	965	1857	0.902
T10	1157	0.16	1.92	609	1720	0.897
T11	1411	0.053	0.755	1052	1585	0.939
T12	1338	0.151	1.709	746	1889	0.89
T13	740	0.193	2.07	383	1149	0.898
T14	882	0.213	2.048	425	1328	0.884
T15	1323	0.129	1.592	747	1800	0.899
T16	873	0.179	1.755	523	1289	0.877
T17	1651	0.089	1.045	1183	2046	0.916
T18	1614	0.135	1.575	937	2208	0.893
T19	799	0.096	1.111	561	1005	0.906
T20	1200	0.151	1.637	711	1693	0.892
T21	1555	0.146	1.631	422	2169	0.898
T22	847	0.185	2.099	422	1311	0.884
T23	1505	0.139	1.69	837	2109	0.897
T24	1245	0.132	1.65	770	1797	0.895
T25	1140	0.133	1.339	724	1487	0.902
T26	774	0.116	1.336	515	1032	0.904
T27	1179	0.077	0.938	884	1437	0.926
T28	743	0.196	2.027	372	1125	0.886
T29	1671	0.089	1.065	1134	2024	0.914
T30	874	0.075	0.847	666	1036	0.935
T31	998	0.17	1.908	684	1491	0.887
T32	996	0.112	1.367	684	1365	0.893
T33	930	0.097	1.151	651	1186	0.917
T34	1190	0.15	1.837	644	1737	0.895

Table S7: Responses of molecular networks for 34 experimental conditions on Feature-Based Molecular Networking workflow on sample *Halidrys siliquosa*.

Analysis of Results (Minitab)

MN Workflow	Sample	Model	R^2	R^2 (adjusted)	R^2 (predicted)	PRESS	S	P
Classical	*Ascidia virginea* 10101	Number of Nodes*	99.88	99.35	95.33	0.0002182	0.0009566	<0.01
		Cluster Co-efficient	89.49	42.18	0	80.2331	0.680872	0.218
		Number of neighbours	85.88	22.34	0	170.753	0.879827	0.378
		Number of Self-loop Nodes*	99.05	94.79	62.65	17433.9	8.58172	<0.01
		Number of Edges*	99.54	97.48	86.15	0.564805	0.558403	<0.01
		Average Cosine score	97.22	84.72	0	0.0123225	0.0075031	<0.01
	Parazoanthus axinellae 10128	Number of Nodes*	99.36	96.5	75.35	38873.7	12.9335	<0.01
		Cluster Co-efficient	83.33	8.32	0	11.7632	0.23887	0.491
		Number of neighbours	94.11	67.58	0	3.85937	0.137489	0.059
		Number of Self-loop Nodes*	99.14	95.27	72.69	12459.8	8.08934	<0.01
		Number of Edges*	98.21	90.14	41.24	192877	31.3114	<0.01
		Average Cosine score*	97.66	87.11	58.51	0.0031096	0.0054111	<0.01
	Halidrys siliquosa 10186	Number of Nodes*	98.93	94.1	56.12	28551867	340.949	<0.01
		Cluster Co-efficient	89.91	44.51	0	0.199414	0.0288213	0.201
		Number of neighbours	98.05	89.26	4.54313	4.54313	0.136196	<0.01
		Number of Self-loop Nodes*	98.02	89.12	21.52	0.0063274	0.0051559	<0.01
		Number of Edges*	99.11	95.09	64.03	0.904868	0.0611518	<0.01
		Average Cosine score	90	45.01	0	0.039424	0.0128541	0.201
Feature-Based	*Ascidia virginea* 10101	Number of Nodes*	99.95	99.75%	98.2	50695.7	14.7346	<0.01
		Cluster Co-efficient*	99.3	96.13	73.19	0.0122798	0.0073247	<0.01
		Number of neighbours*	99.61	97.83	86.24	0.763922	0.0604292	<0.01
		Number of Self-loop Nodes*	99.96	99.77	98.55	30585.9	98.55	<0.01
		Number of Edges*	99.78	98.8	91.52	278493	91.5	<0.01
		Average Cosine score*	99.71	98.41	93.73	0.000521	0.002	<0.01
	Parazoanthus axinellae 10128	Number of Nodes	94.46	69.55	0	1.87E+13	276342	0.05
		Cluster Co-efficient*	98.26	90.45	30.52	278.027	1.07589	<0.01
		Number of neighbours	94.55	70.01	0	7.60682	0.175936	0.049
		Number of Self-loop Nodes*	98.51	91.8	40.63	1697325	84.2852	<0.01
		Number of Edges*	99.62	97.9	86.33	0.0000484	0.000475	<0.01
		Average Cosine score	96.94	83.17	0	0.0112007	0.0068828	0.011
	Halidrys siliquosa 10186	Number of Nodes*	99.85	99.19	94.57	0	0	<0.01
		Cluster Co-efficient*	98.27	90.5	34.11	226.096	0.994139	<0.01
		Number of neighbours*	99.87	99.29	94.72	0.245578	0.0316518	<0.01
		Number of Self-loop Nodes	96.56	81.08	0	5.23E+12	147923	0.015
		Number of Edges*	99.47	97.1	82.64	116.255	0.767847	<0.01
		Average Cosine score*	99.25	95.87	70.01	0.0044473	0.0043076	<0.01

Table S8: R^2, R^2 (predicted), R^2 (adjusted), PRESS, S and P statistical values of response models for three samples and two workflows. Models included in summary analysis are indicated with an asterisk. Models highlighted in red were excluded from final analysis because non-significant (R^2<20).

DOE reports

Classical Molecular Networking

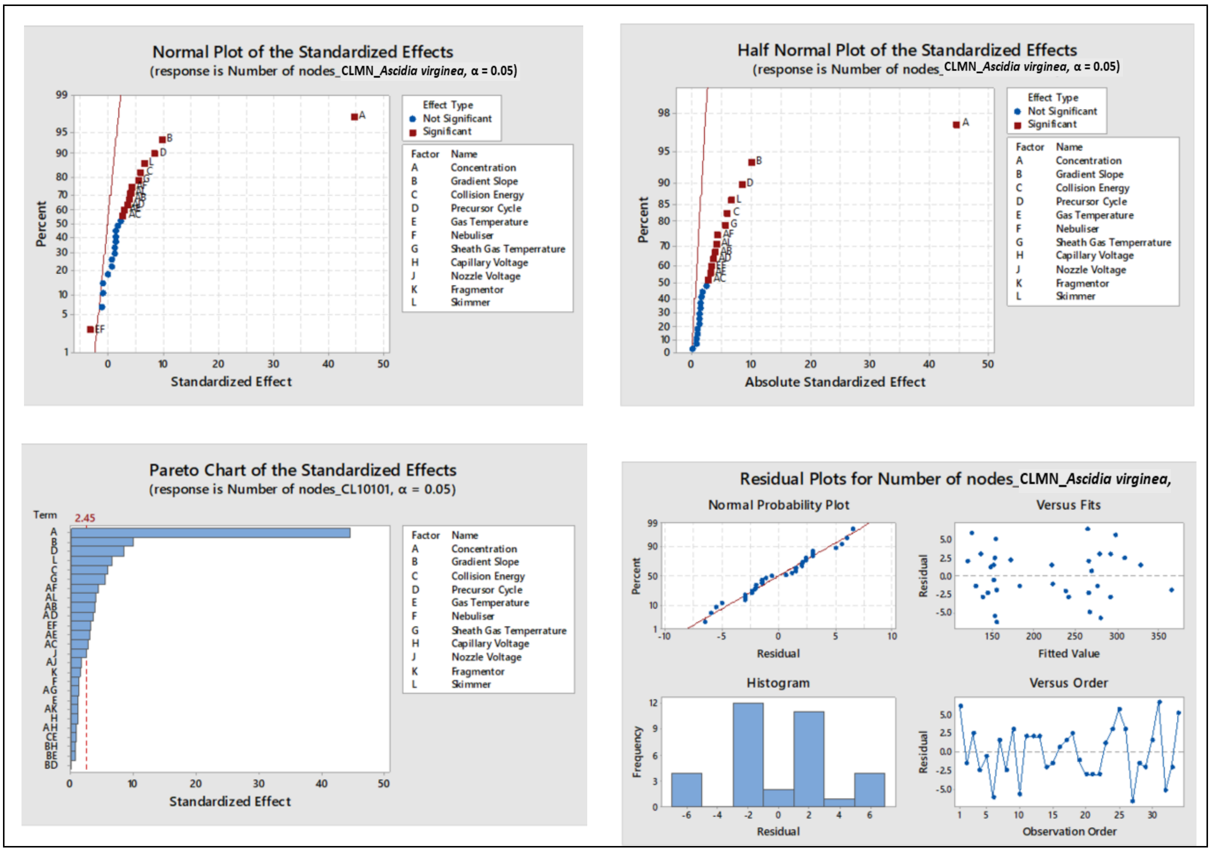

Figure S4: DOE reports for number of node model in Classical Molecular Networking of *Ascidia virginea*, normal plot of standardized effects (top left), half normal plot of standardized effects (top right), pareto chart of standardized effects (bottom left), and residual plots (bottom right).

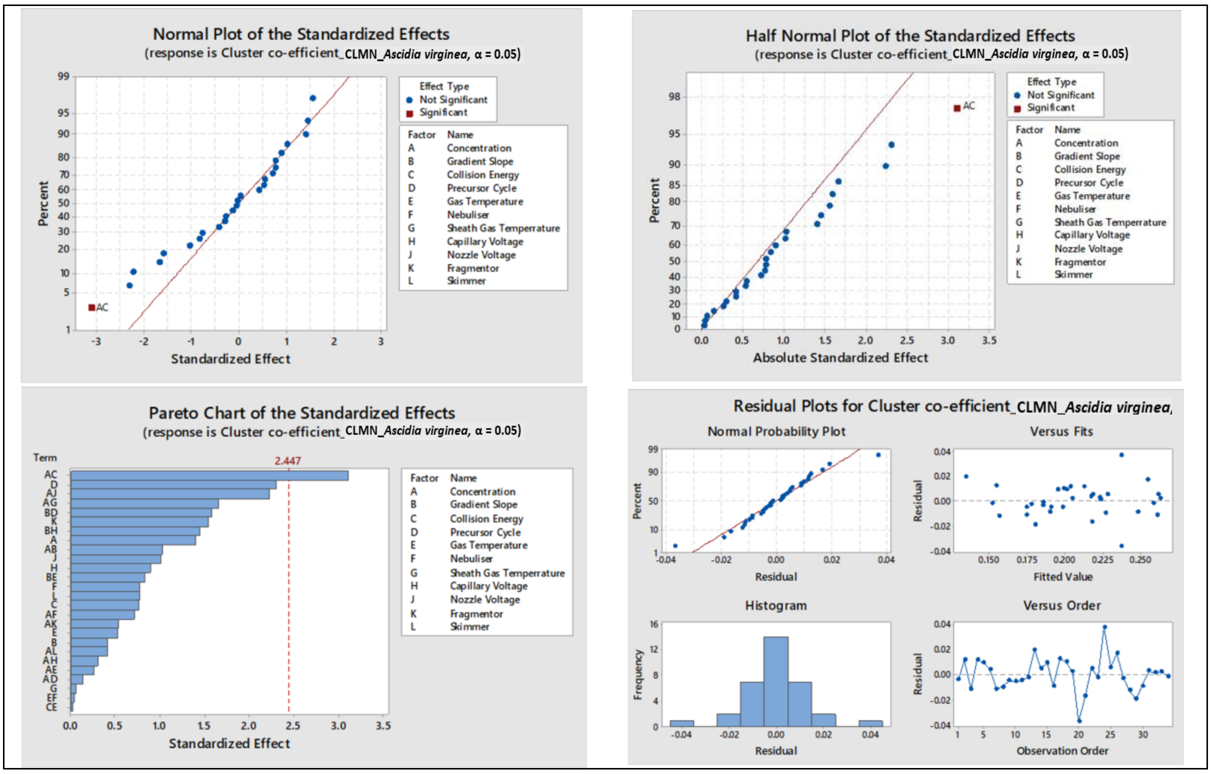

Figure S5: DOE reports for cluster co-efficient model in Classical Molecular Networking of *Ascidia virginea*, normal plot of standardized effects (top left), half normal plot of standardized effects (top right), pareto chart of standardized effects (bottom left), and residual plots (bottom right).

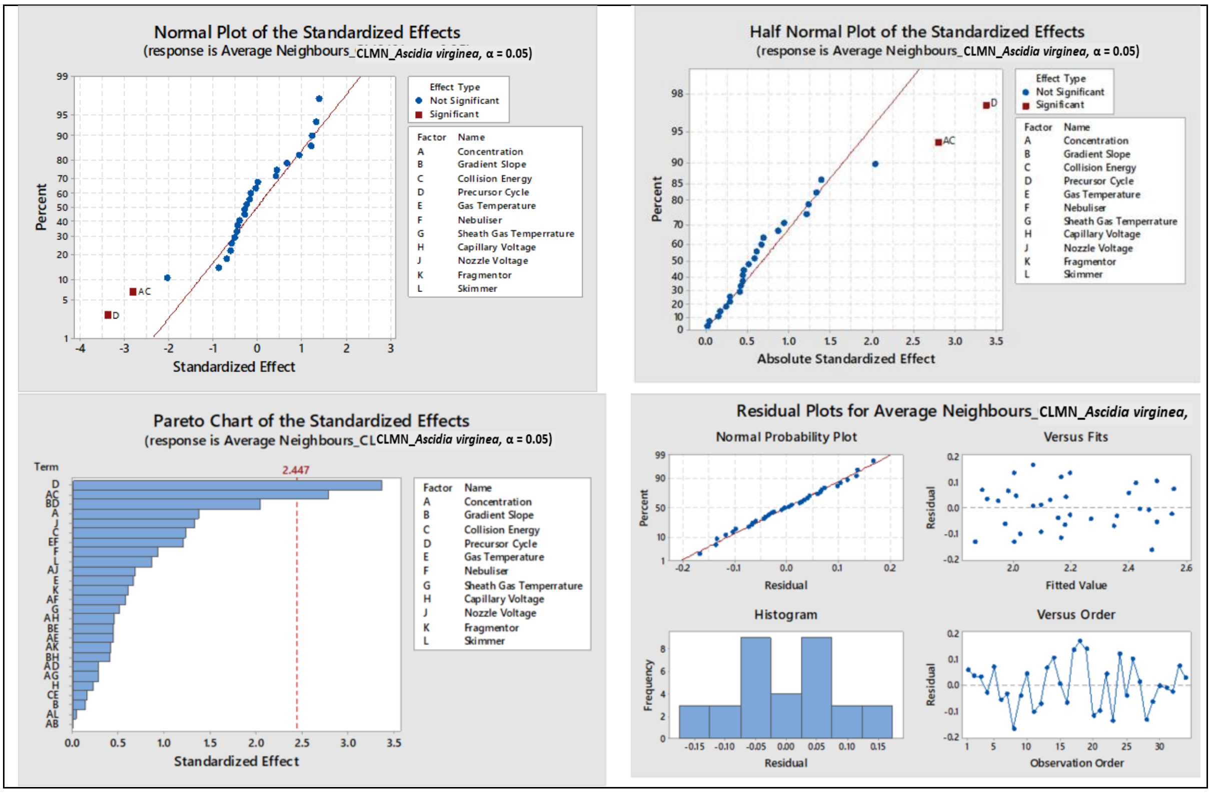

Figure S6: DOE reports for average number of neighbours model in Classical Molecular Networking of *Ascidia virginea*, normal plot of standardized effects (top left), half normal plot of standardized effects (top right), pareto chart of standardized effects (bottom left), and residual plots (bottom right).

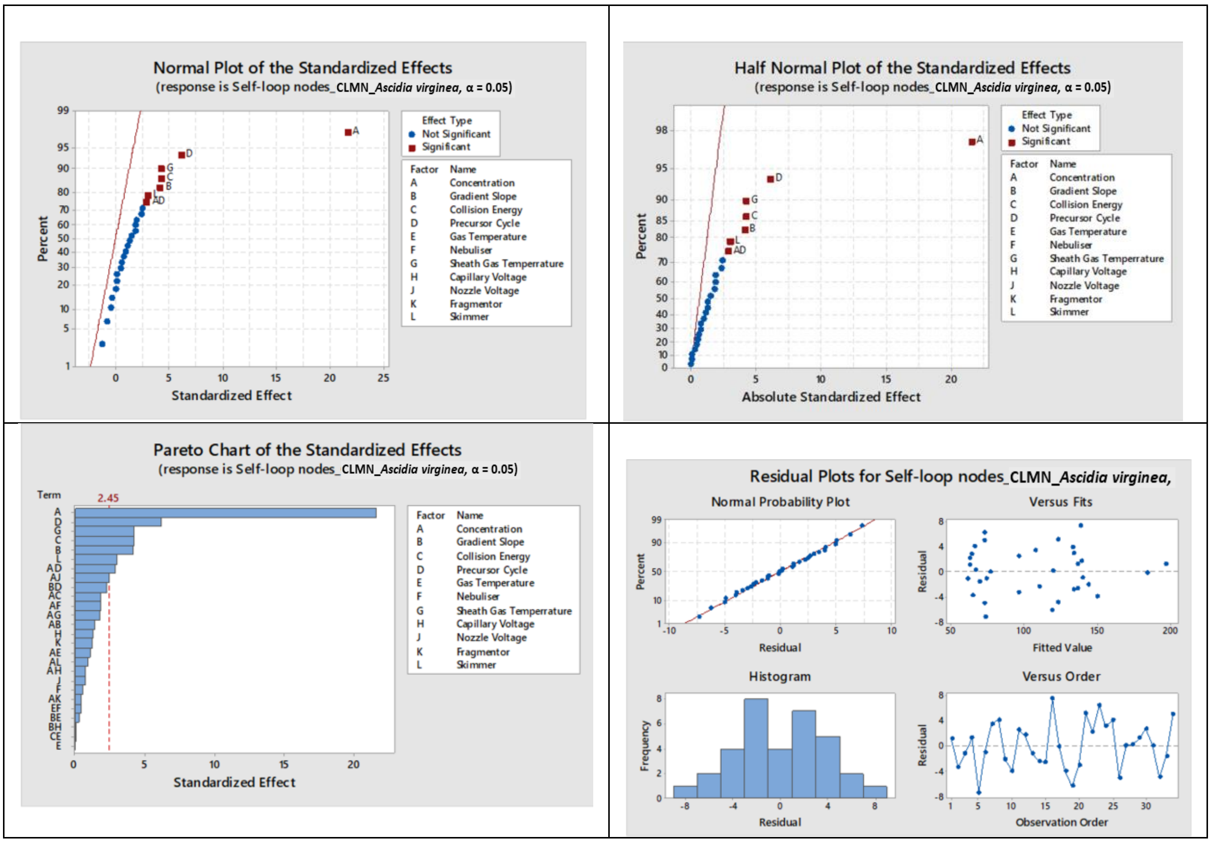

Figure S7: DOE reports for number of self-loop nodes model in Classical Molecular Networking of *Ascidia virginea*, normal plot of standardized effects (top left), half normal plot of standardized effects (top right), pareto chart of standardized effects (bottom left), and residual plots (bottom right).

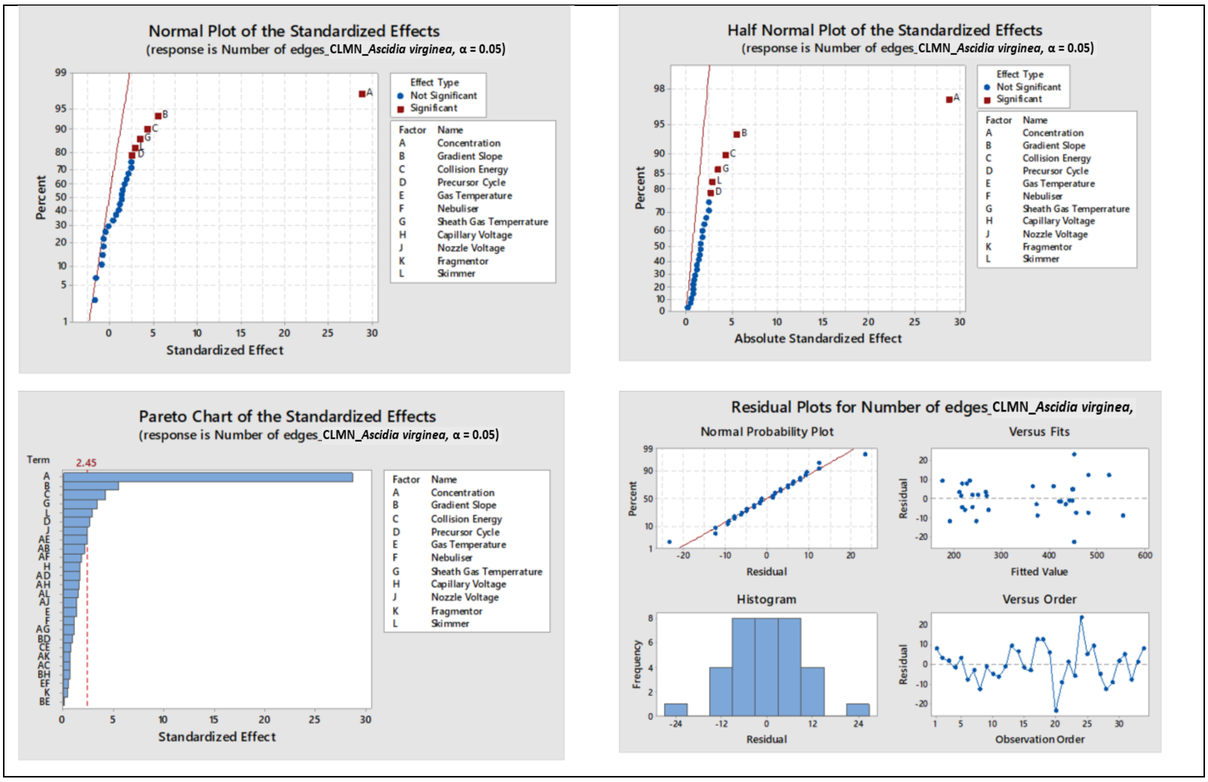

Figure S8: DOE reports for number of edges model in Classical Molecular Networking of *Ascidia virginea*, normal plot of standardized effects (top left), half normal plot of standardized effects (top right), pareto chart of standardized effects (bottom left), and residual plots (bottom right).

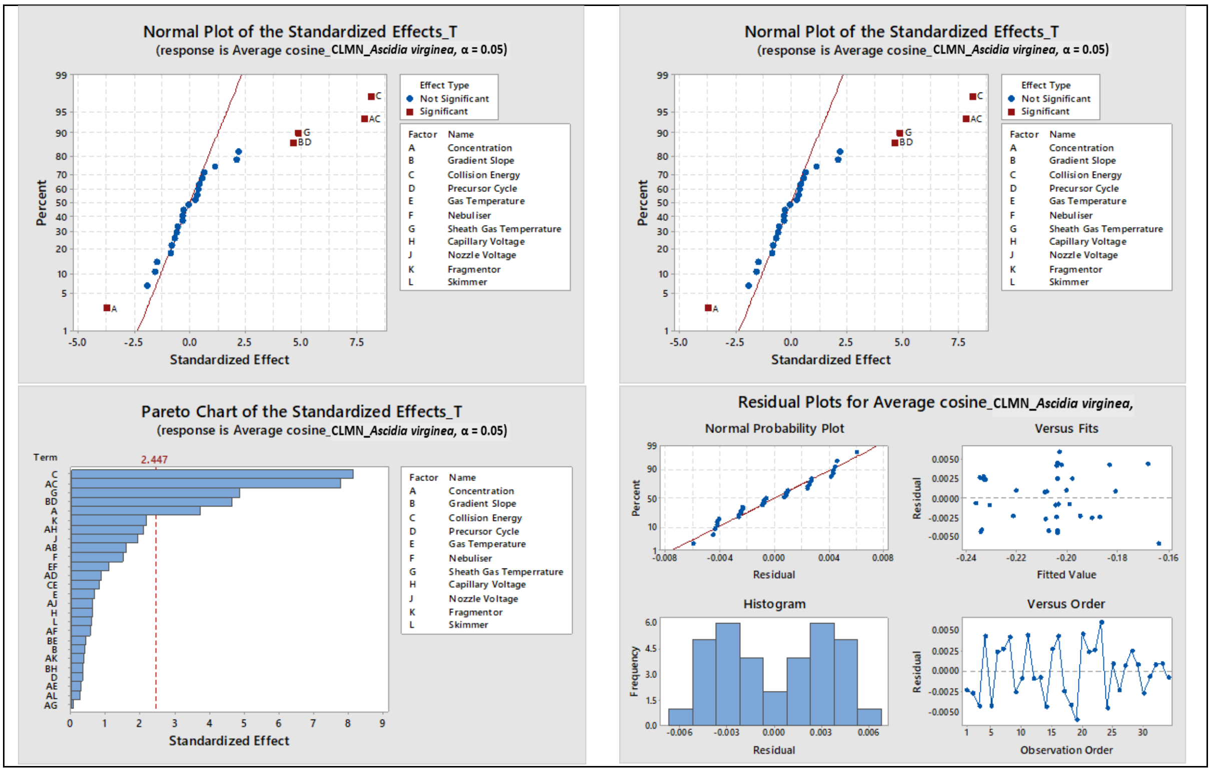

Figure S9: DOE reports for average cosine score model in Classical Molecular Networking of *Ascidia virginea*, normal plot of standardized effects (top left), half normal plot of standardized effects (top right), pareto chart of standardized effects (bottom left), and residual plots (bottom right).

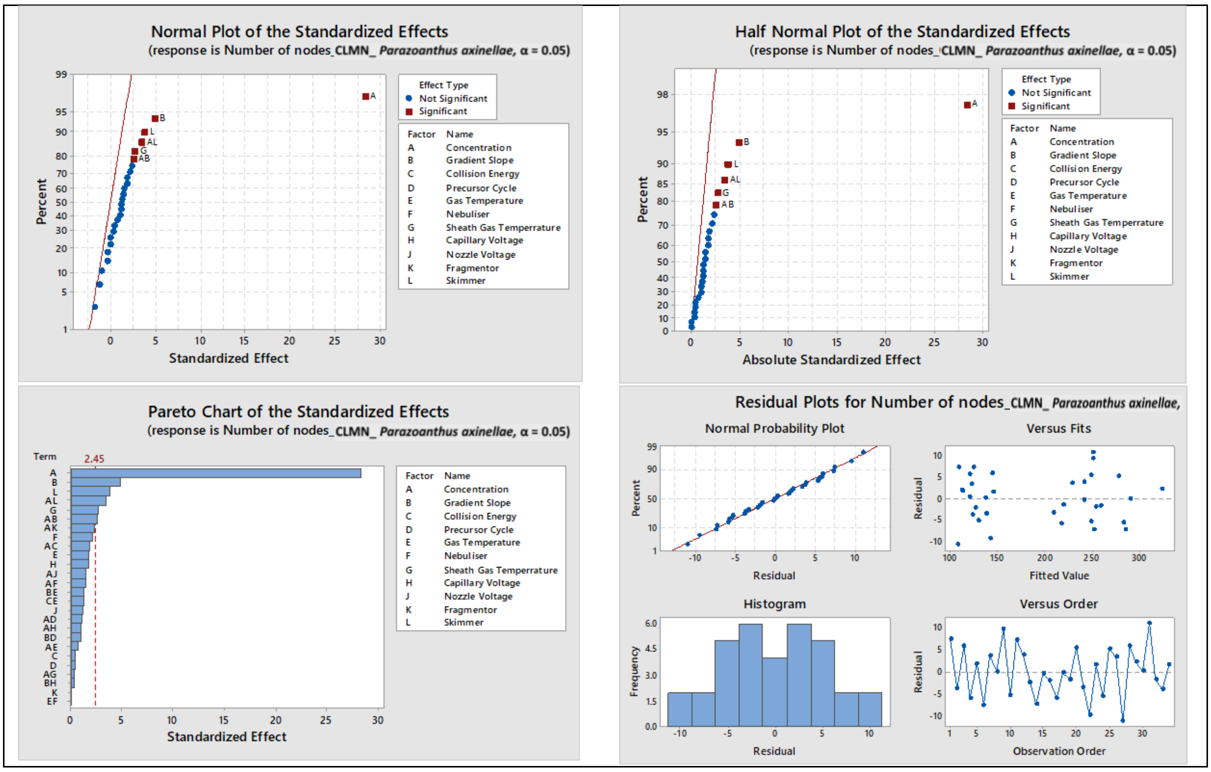

Figure S10: DOE reports for number of nodes model in Classical Molecular Networking of *Parazoanthus axinellae*, normal plot of standardized effects (top left), half normal plot of standardized effects (top right), pareto chart of standardized effects (bottom left), and residual plots (bottom right).

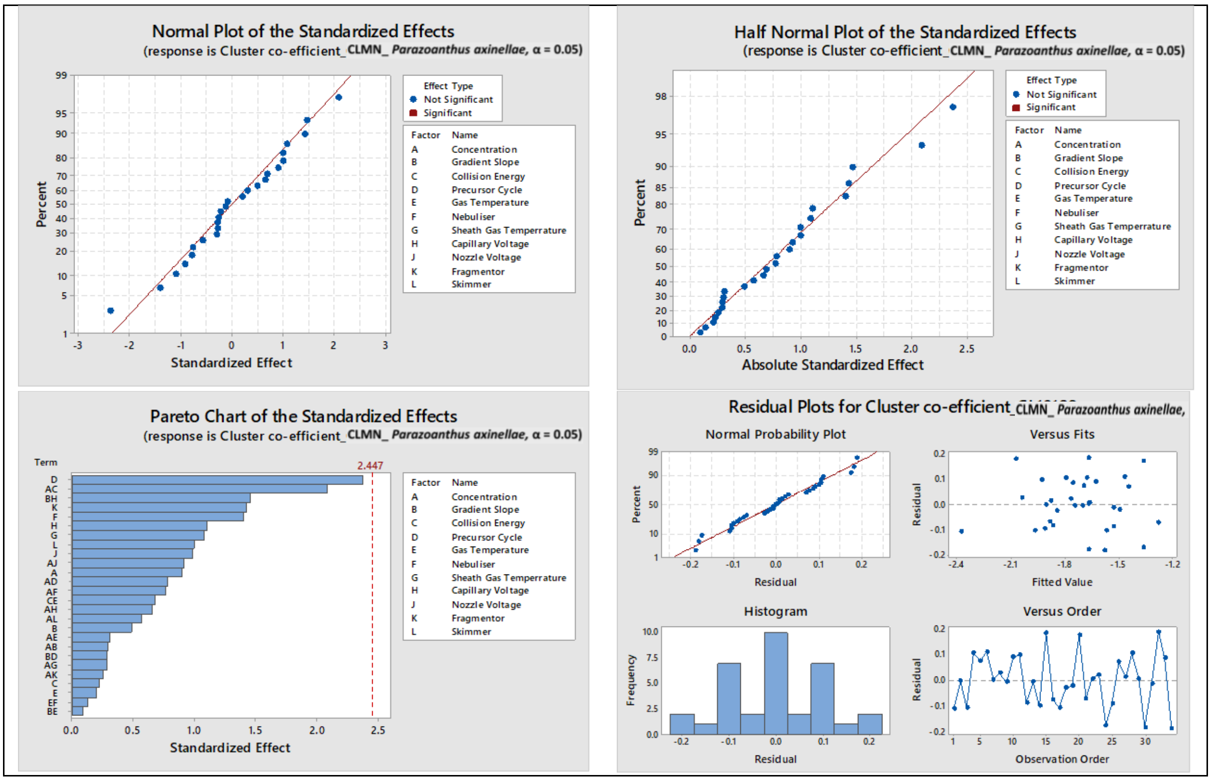

Figure S11: DOE reports for cluster co-efficient model in Classical Molecular Networking of *Parazoanthus axinellae*, normal plot of standardized effects (top left), half normal plot of standardized effects (top right), pareto chart of standardized effects (bottom left), and residual plots (bottom right).

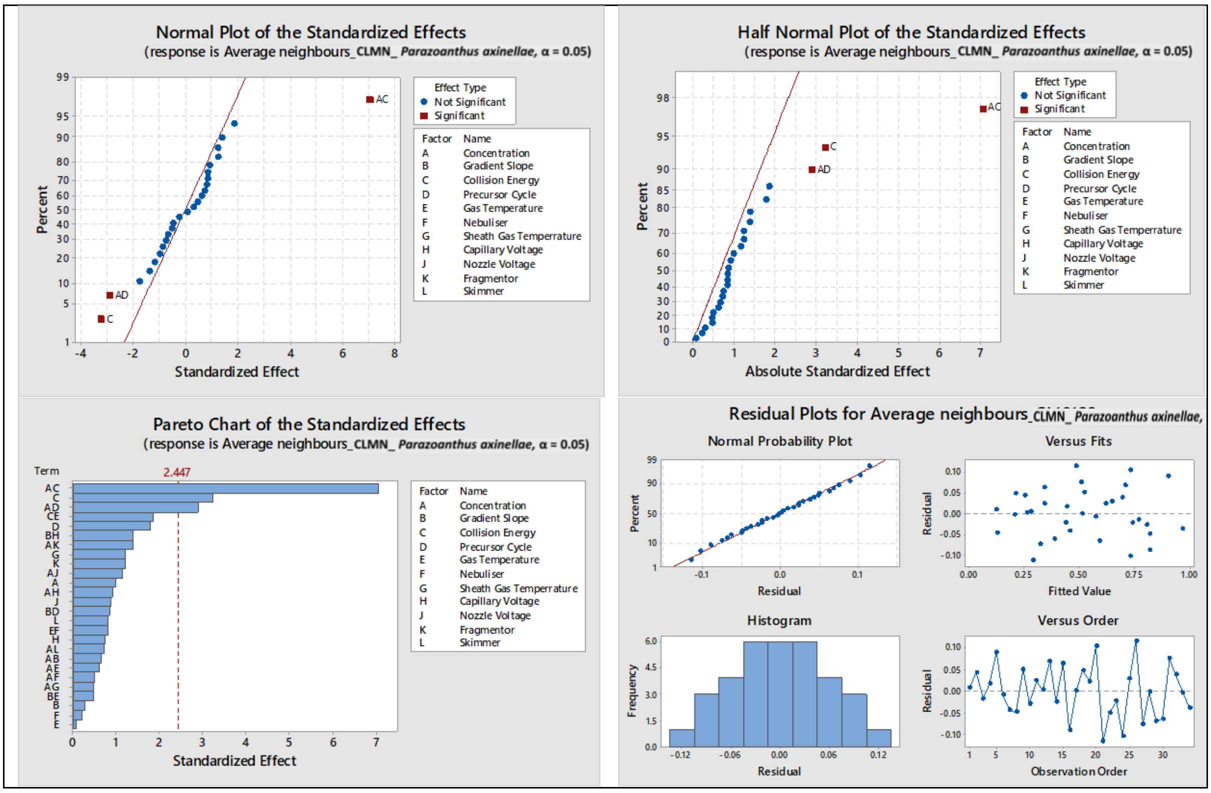

Figure S12: DOE reports for average number of neighbours model in Classical Molecular Networking of *Parazoanthus axinellae*, normal plot of standardized effects (top left), half normal plot of standardized effects (top right), pareto chart of standardized effects (bottom left), and residual plots (bottom right).

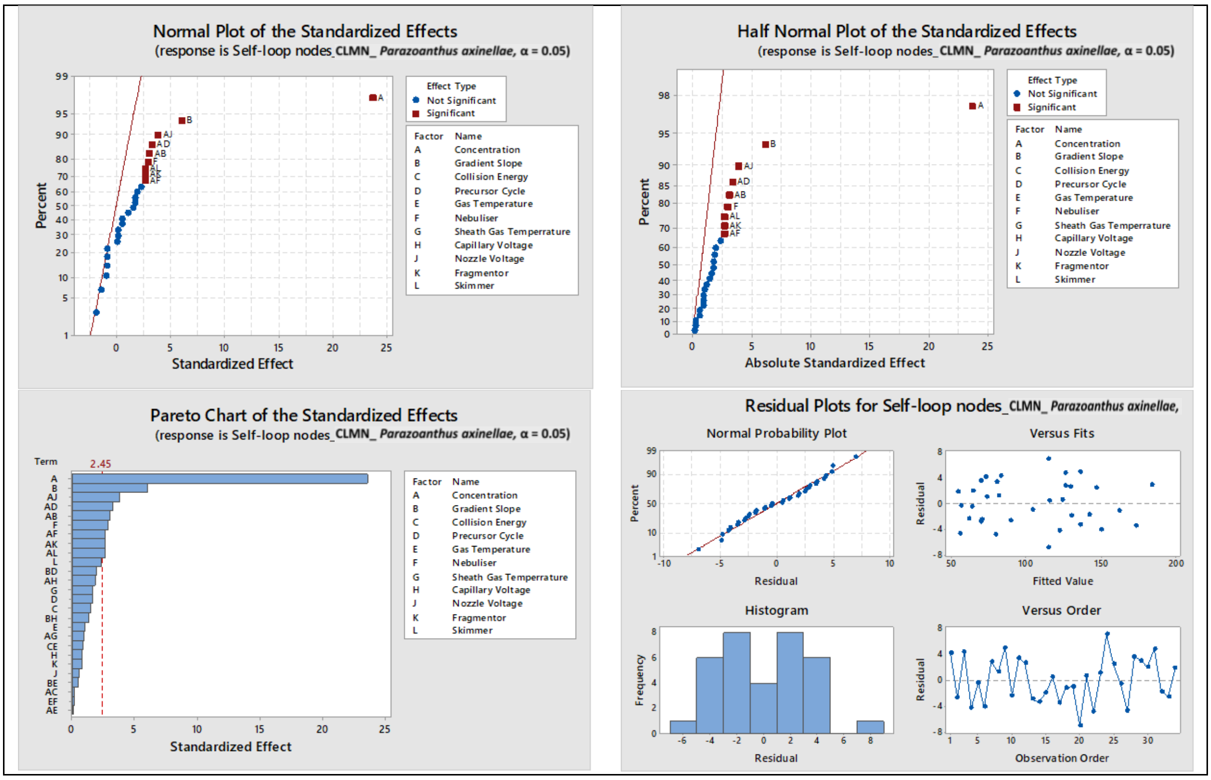

Figure S13: DOE reports for number of self-loop nodes model in Classical Molecular Networking of *Parazoanthus axinellae*, normal plot of standardized effects (top left), half normal plot of standardized effects (top right), pareto chart of standardized effects (bottom left), and residual plots (bottom right).

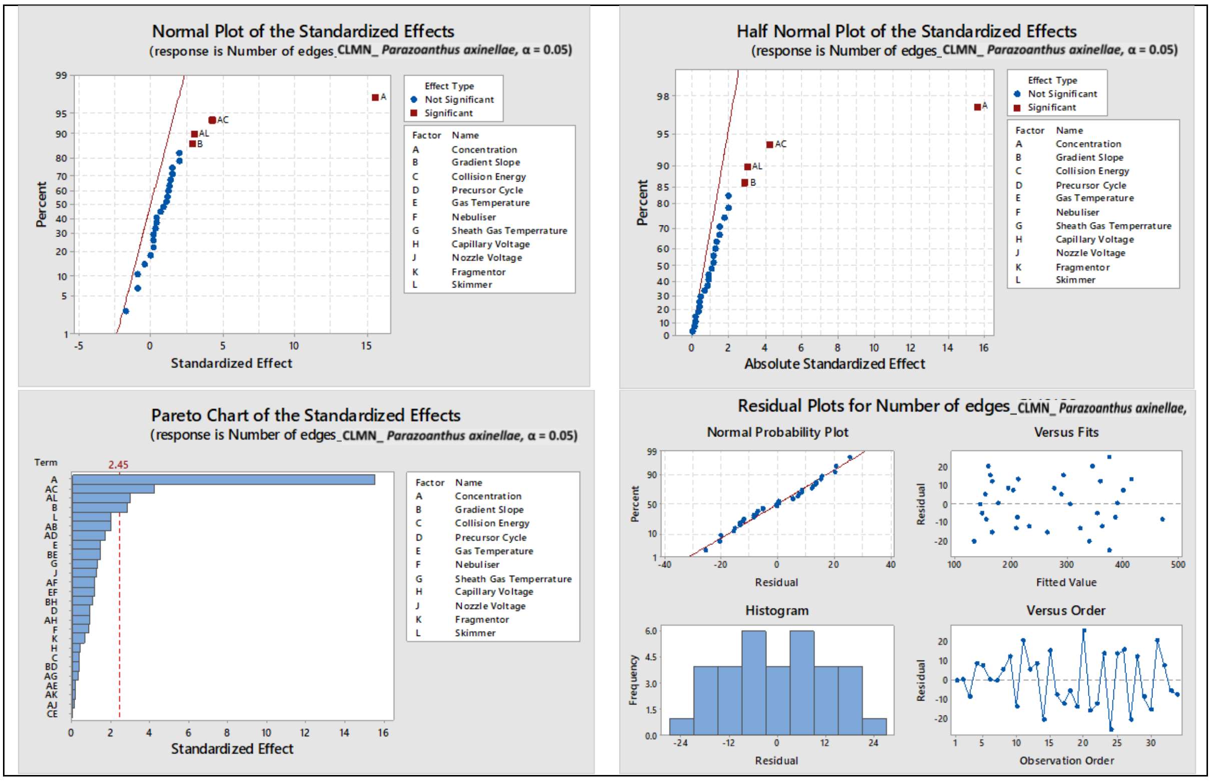

Figure S14: DOE reports for number of edges model in Classical Molecular Networking of *Parazoanthus axinellae*, normal plot of standardized effects (top left), half normal plot of standardized effects (top right), pareto chart of standardized effects (bottom left), and residual plots (bottom right).

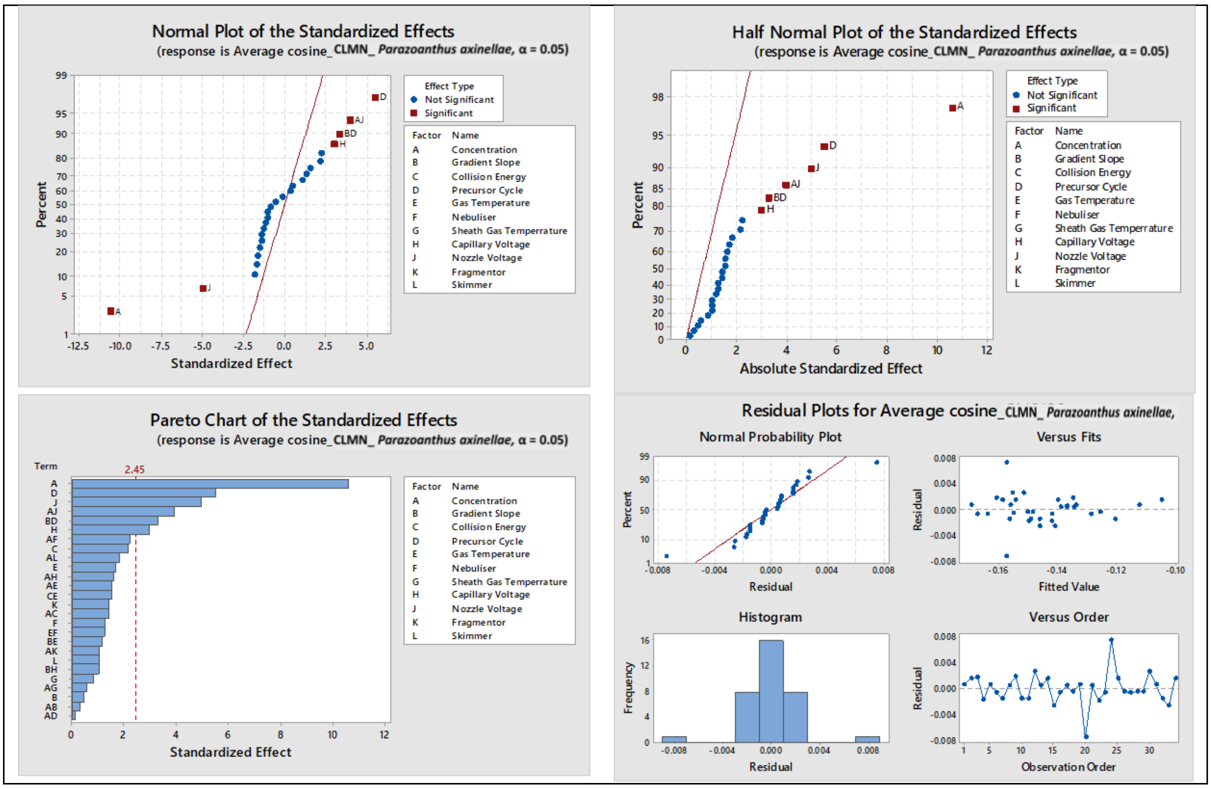

Figure S15: DOE reports for average cosine score model in Classical Molecular Networking of *Parazoanthus axinellae*, normal plot of standardized effects (top left), half normal plot of standardized effects (top right), pareto chart of standardized effects (bottom left), and residual plots (bottom right).

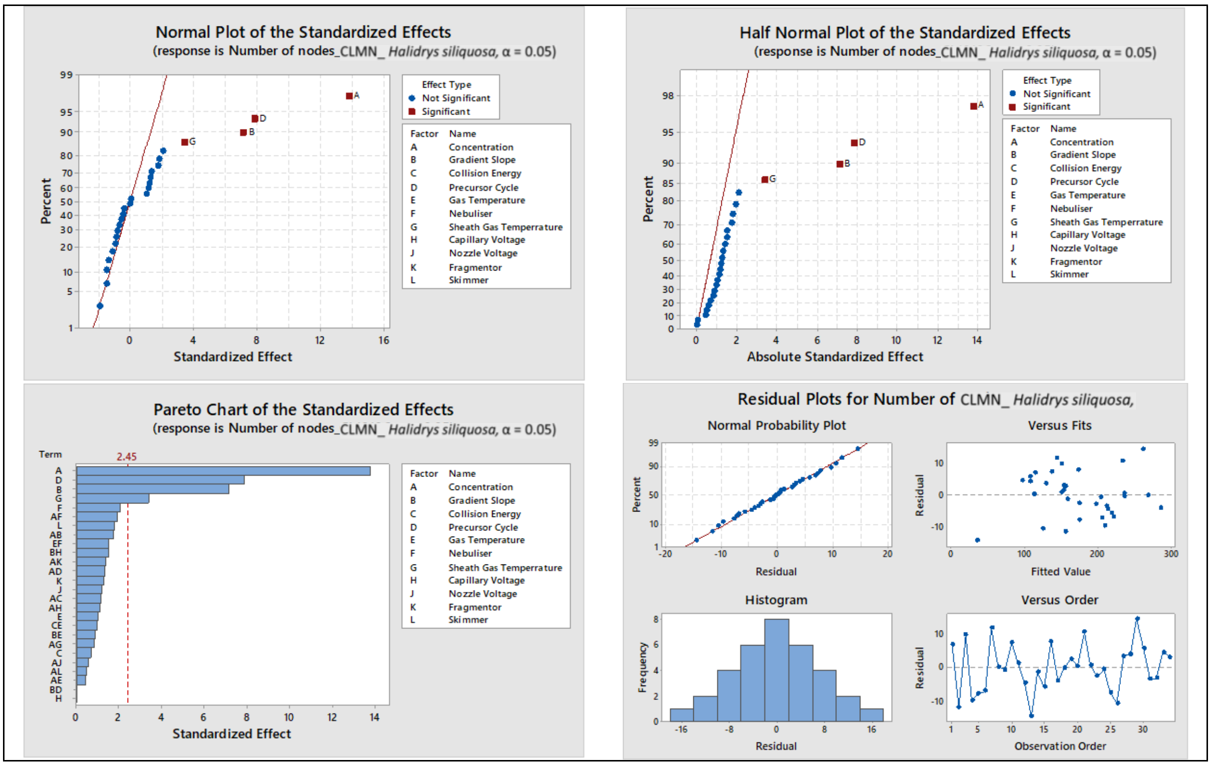

Figure S16: DOE reports for number of nodes model in Classical Molecular Networking of *Halidrys siliquosa*, normal plot of standardized effects (top left), half normal plot of standardized effects (top right), pareto chart of standardized effects (bottom left), and residual plots (bottom right).

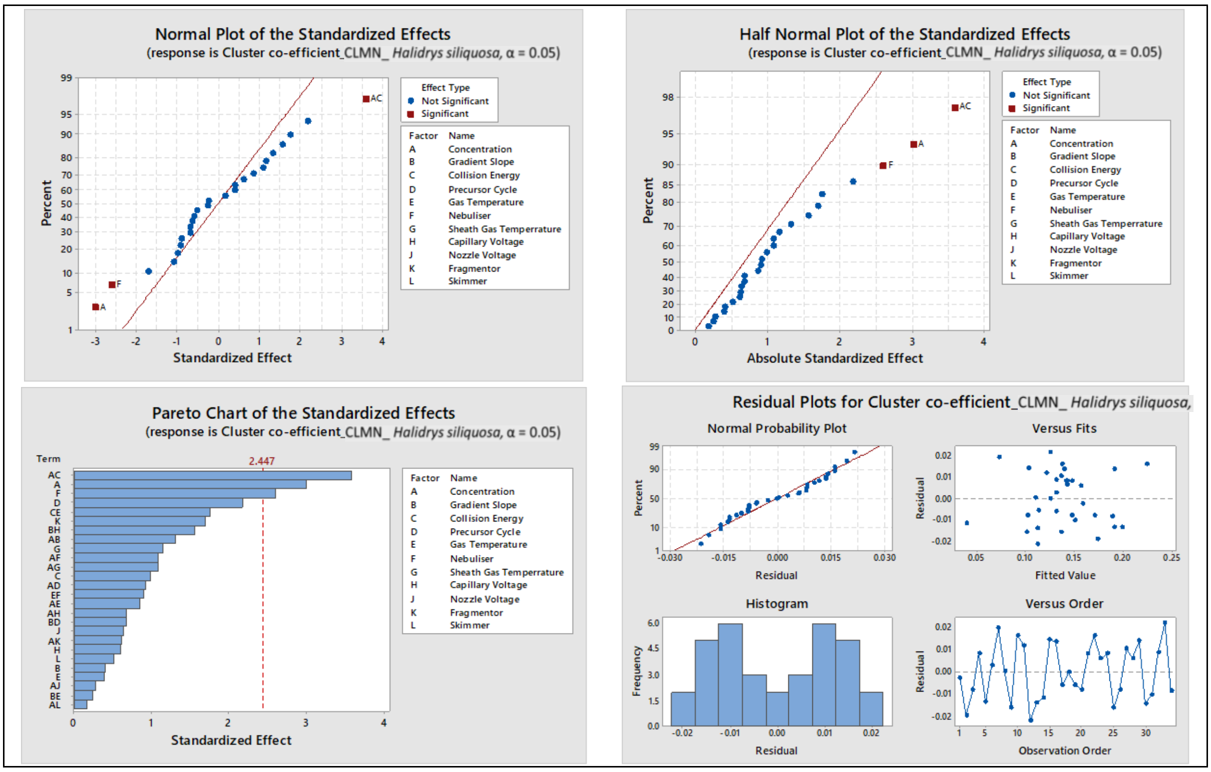

Figure S17: DOE reports for cluster co-efficient model in Classical Molecular Networking of *Halidrys siliquosa*, normal plot of standardized effects (top left), half normal plot of standardized effects (top right), pareto chart of standardized effects (bottom left), and residual plots (bottom right).

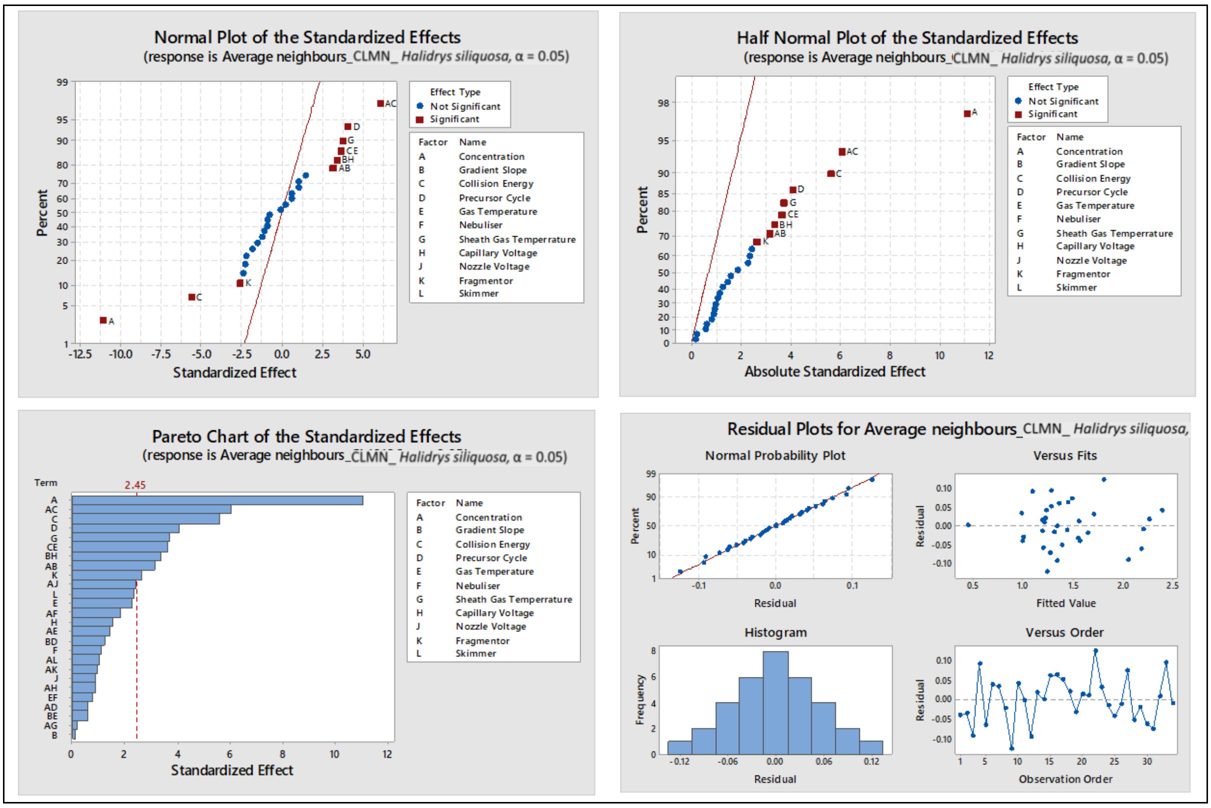

Figure S18: DOE reports for average number of neighbours model in Classical Molecular Networking of *Halidrys siliquosa*, normal plot of standardized effects (top left), half normal plot of standardized effects (top right), pareto chart of standardized effects (bottom left), and residual plots (bottom right).

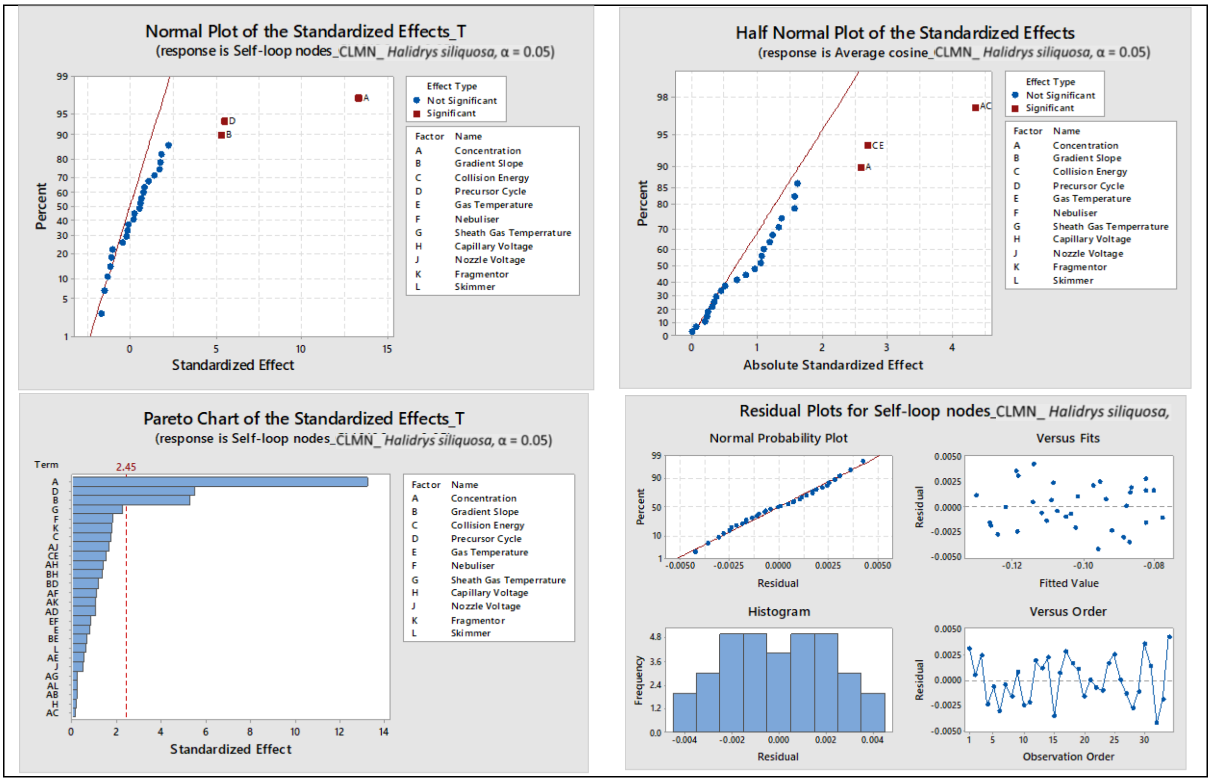

Figure S19: DOE reports for number of self-loop nodes model in Classical Molecular Networking of *Halidrys siliquosa*, normal plot of standardized effects (top left), half normal plot of standardized effects (top right), pareto chart of standardized effects (bottom left), and residual plots (bottom right).

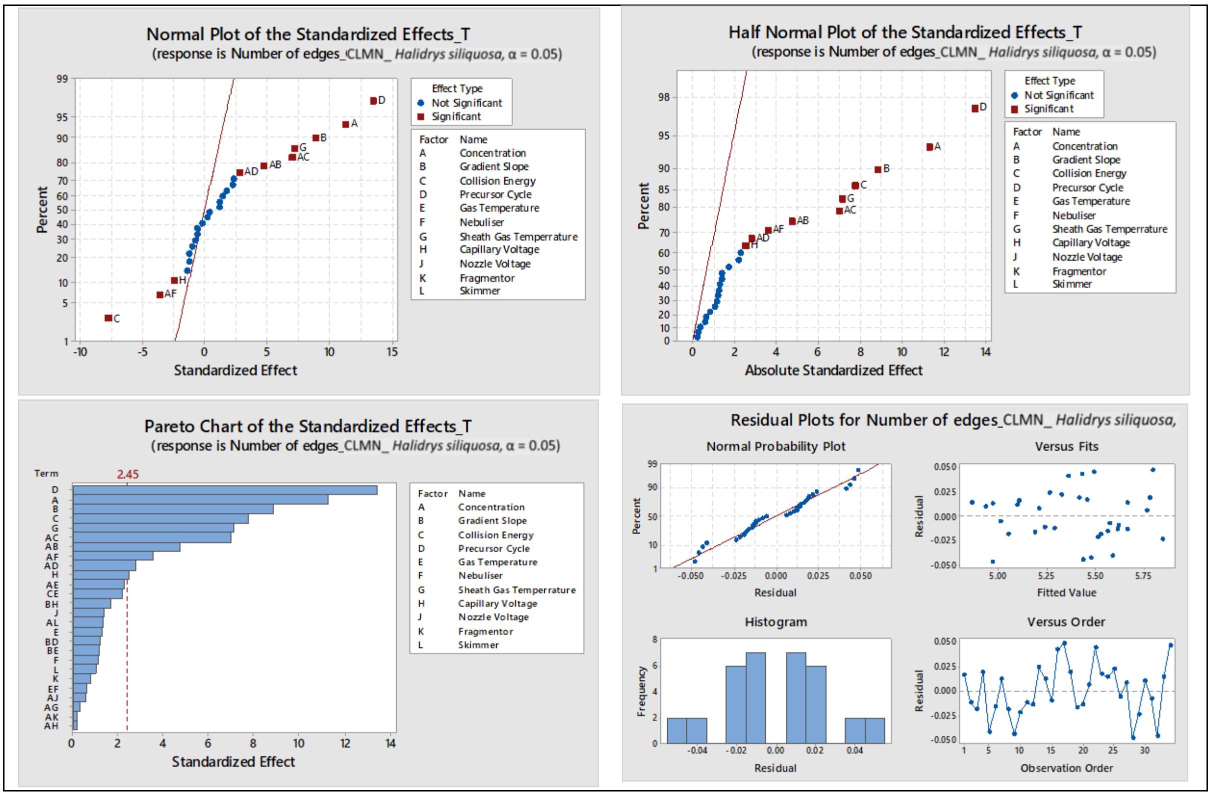

Figure S20: DOE reports for number of nodes model in Classical Molecular Networking of *Halidrys siliquosa*, normal plot of standardized effects (top left), half normal plot of standardized effects (top right), pareto chart of standardized effects (bottom left), and residual plots (bottom right).

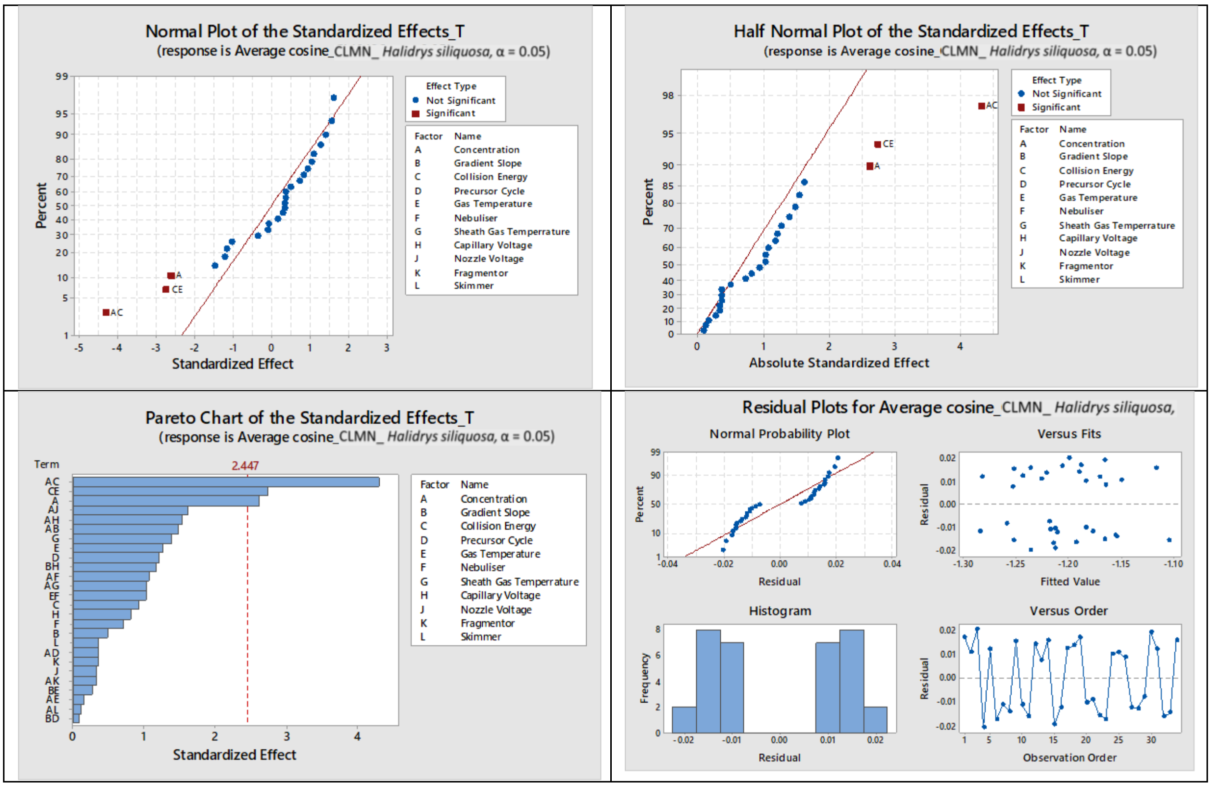

Figure S21: DOE reports for average cosine model in Classical Molecular Networking of *Halidrys siliquosa*, normal plot of standardized effects (top left), half normal plot of standardized effects (top right), pareto chart of standardized effects (bottom left), and residual plots (bottom right).

Feature-Based Molecular Networking

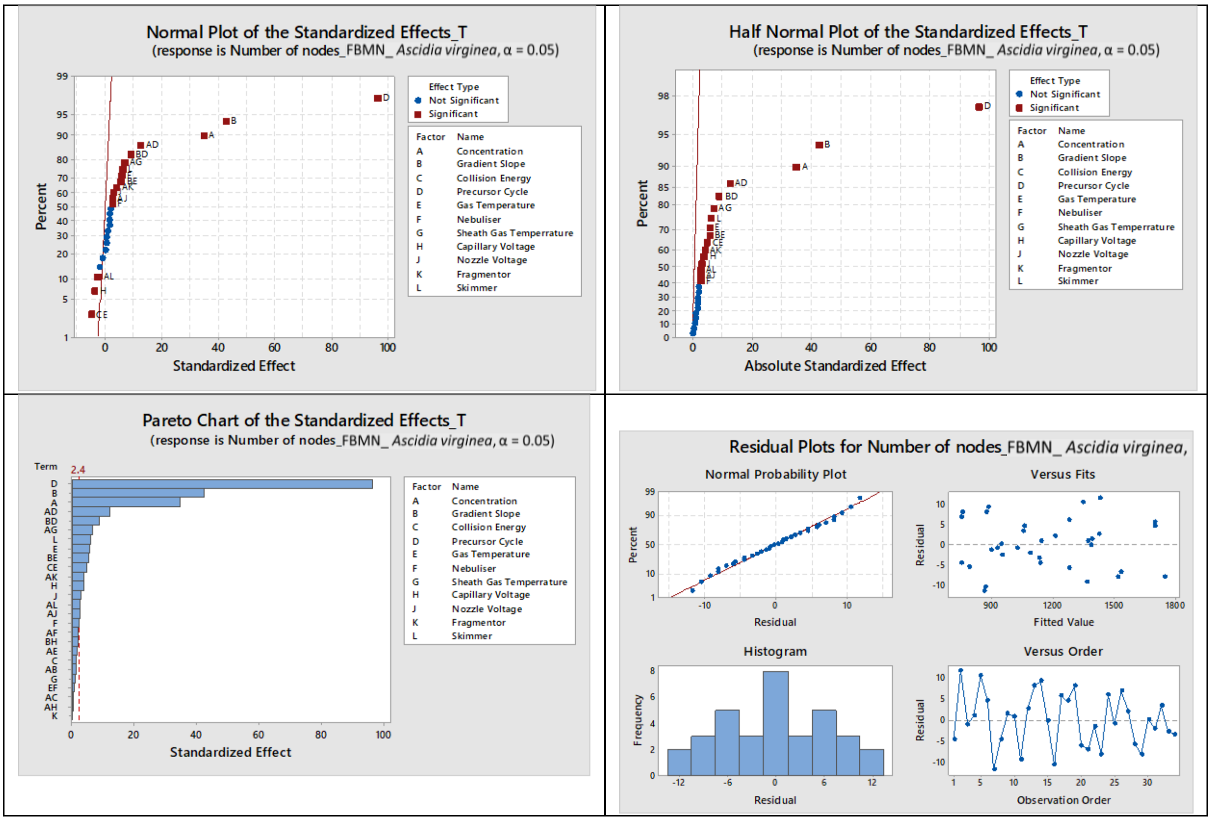

Figure S22: DOE reports for number of node model in Feature-Based Molecular Networking of *Ascidia virginea*, normal plot of standardized effects (top left), half normal plot of standardized effects (top right), pareto chart of standardized effects (bottom left), and residual plots (bottom right).

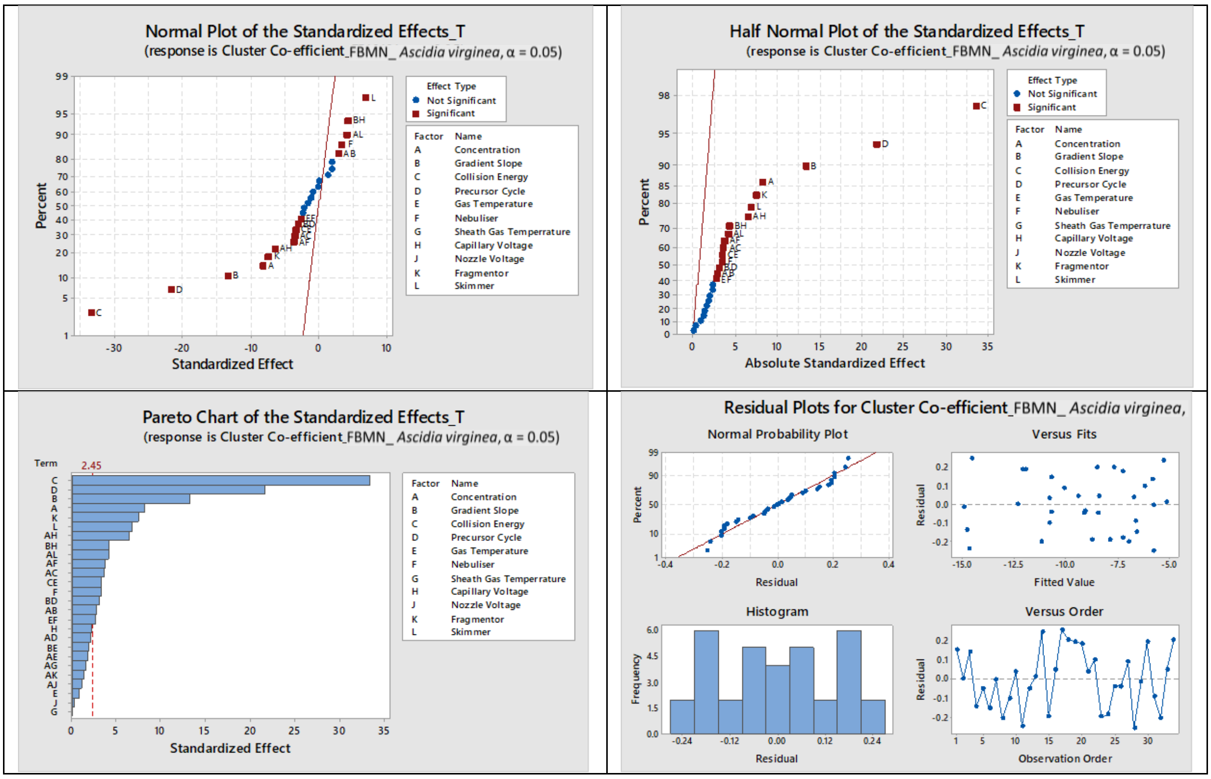

Figure S23: DOE reports for cluster co-efficient model in Feature-Based Molecular Networking of *Ascidia virginea*, normal plot of standardized effects (top left), half normal plot of standardized effects (top right), pareto chart of standardized effects (bottom left), and residual plots (bottom right).

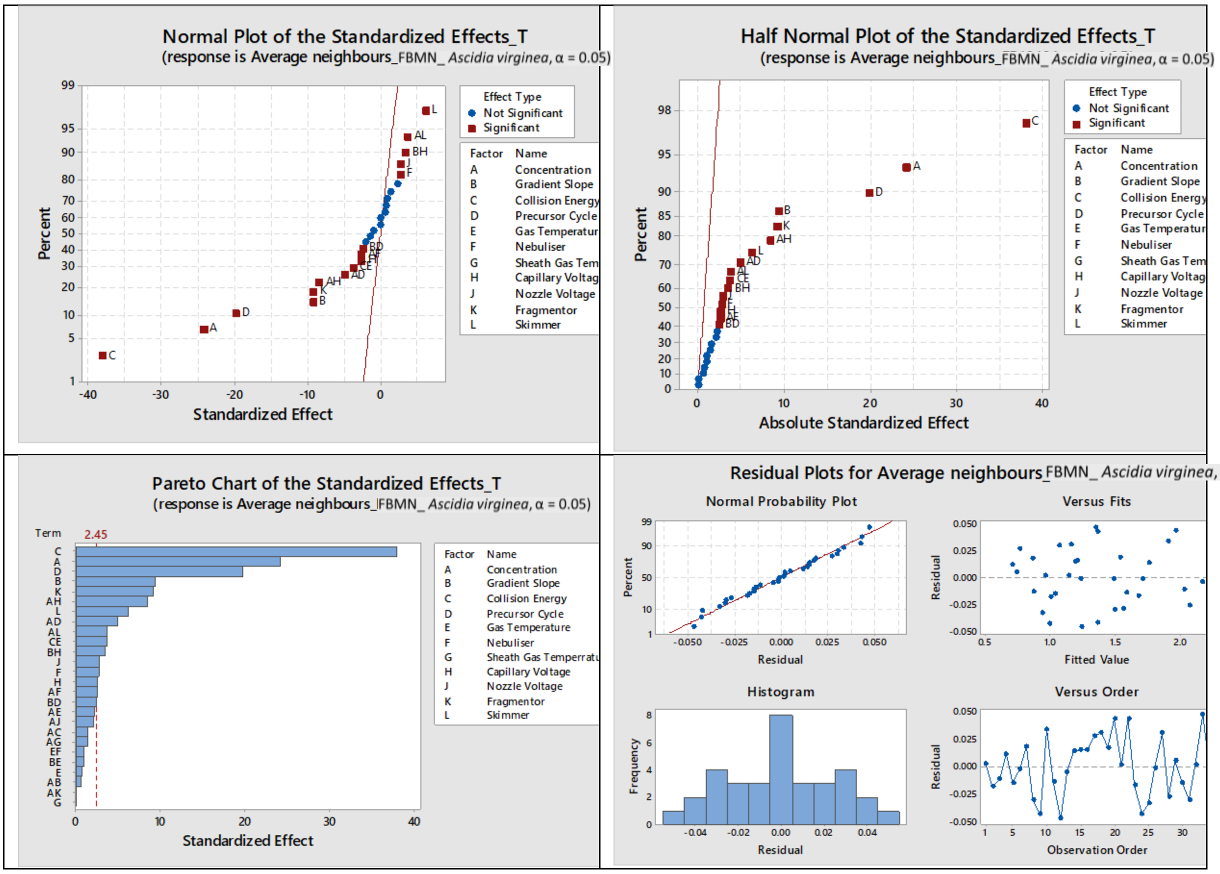

Figure S24: DOE reports for average number of neighbours model in Feature-Based Molecular Networking of *Ascidia virginea*, normal plot of standardized effects (top left), half normal plot of standardized effects (top right), pareto chart of standardized effects (bottom left), and residual plots (bottom right).

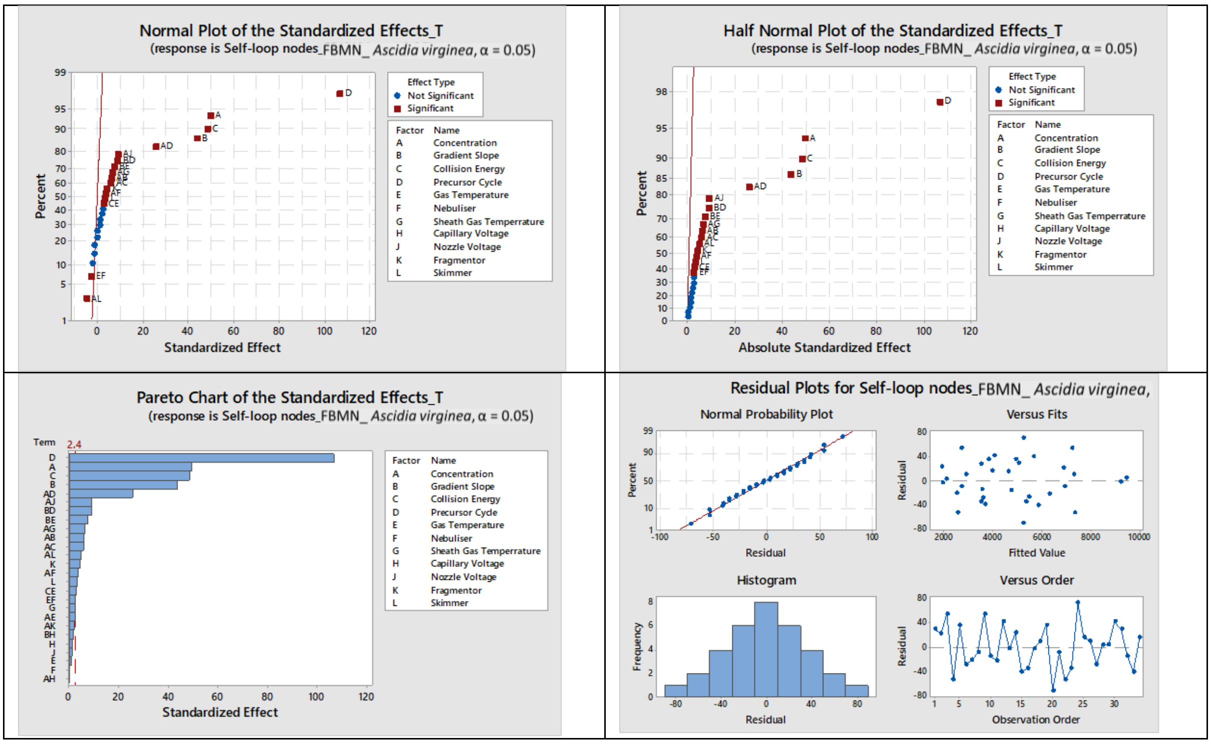

Figure S25: DOE reports for number of self-loop nodes model in Feature-Based Molecular Networking of *Ascidia virginea*, normal plot of standardized effects (top left), half normal plot of standardized effects (top right), pareto chart of standardized effects (bottom left), and residual plots (bottom right).

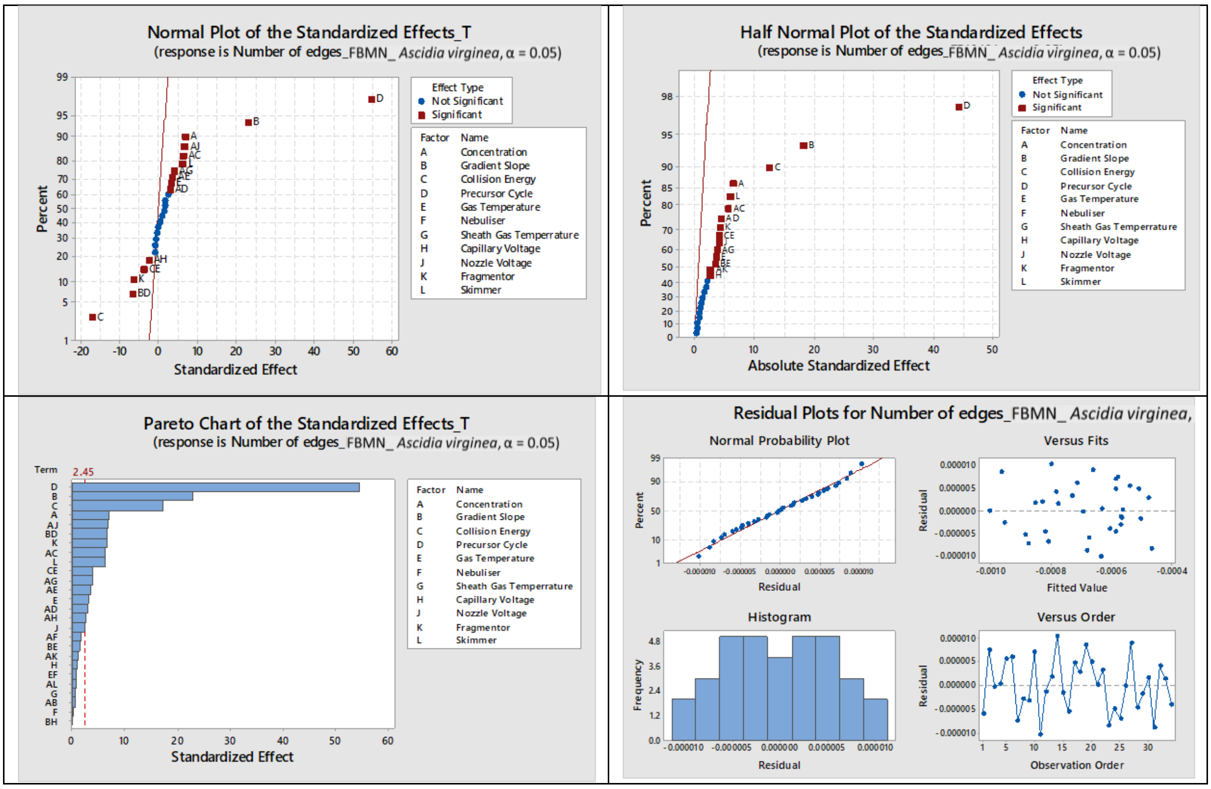

Figure S26: DOE reports for number of edges model in Feature-Based Molecular Networking of *Ascidia virginea*, normal plot of standardized effects (top left), half normal plot of standardized effects (top right), pareto chart of standardized effects (bottom left), and residual plots (bottom right).

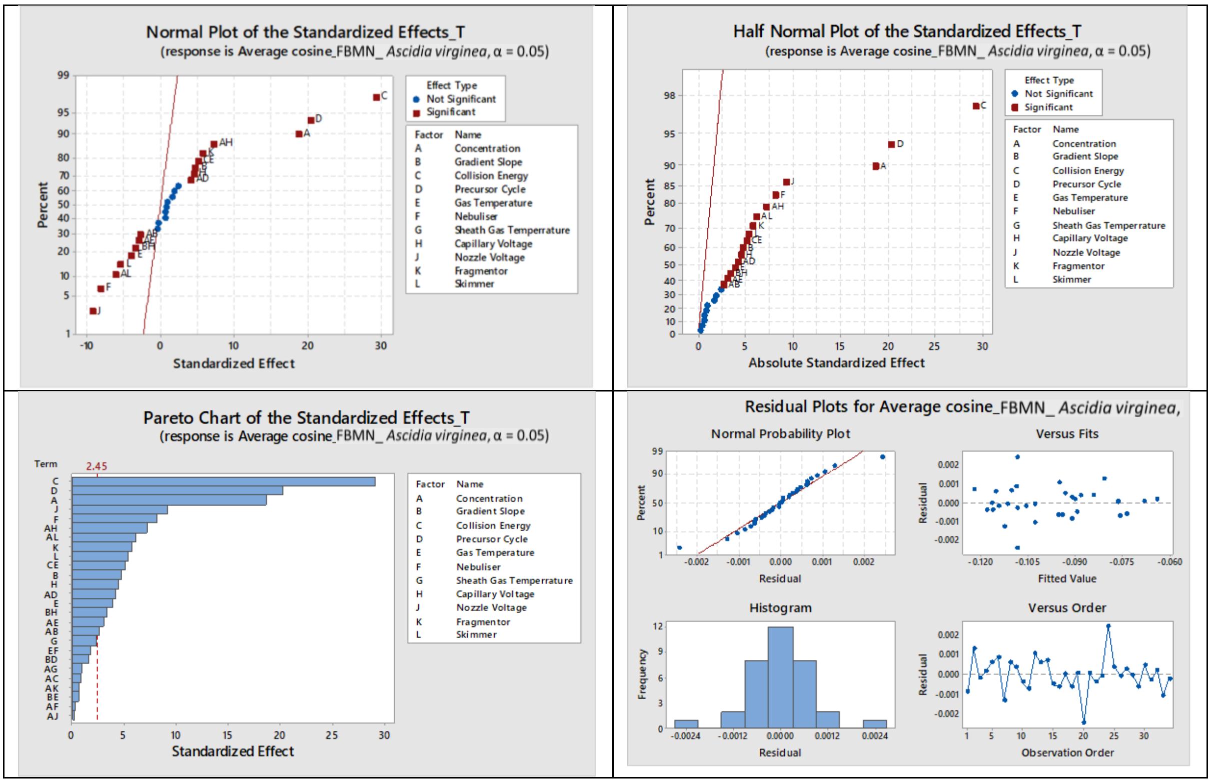

Figure S27: DOE reports for average cosine score model in Feature-Based Molecular Networking of *Ascidia virginea*, normal plot of standardized effects (top left), half normal plot of standardized effects (top right), pareto chart of standardized effects (bottom left), and residual plots (bottom right).

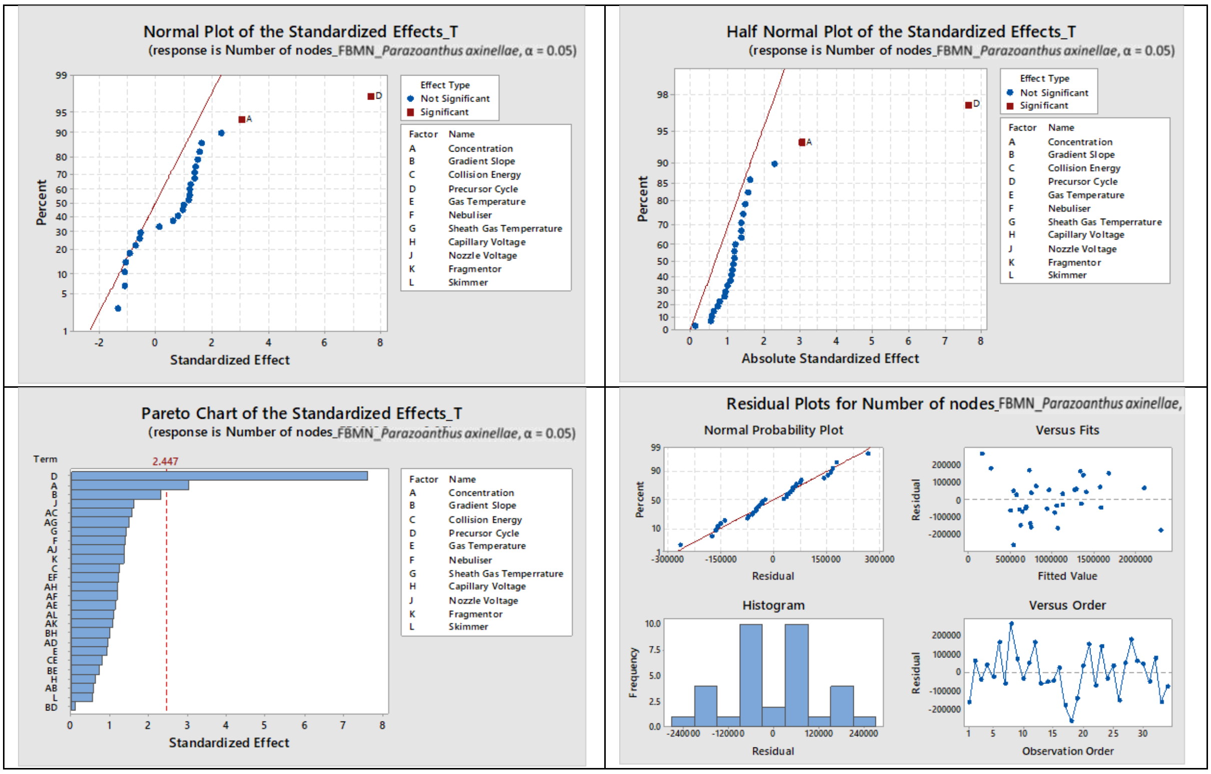

Figure S28: DOE reports for number of node model in Feature-Based Molecular Networking of *Parazoanthus axinellae*, normal plot of standardized effects (top left), half normal plot of standardized effects (top right), pareto chart of standardized effects (bottom left), and residual plots (bottom right).

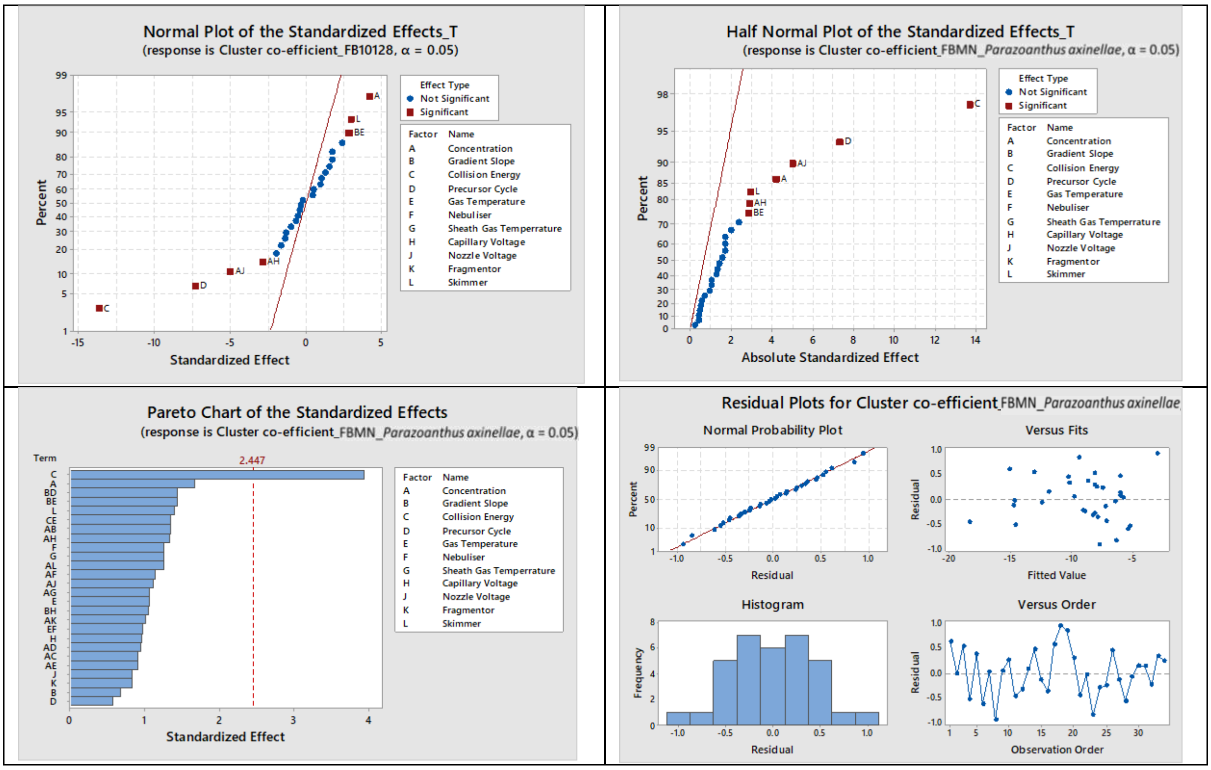

Figure S29: DOE reports for cluster co-efficient model in Feature-Based Molecular Networking of *Parazoanthus axinellae*, normal plot of standardized effects (top left), half normal plot of standardized effects (top right), pareto chart of standardized effects (bottom left), and residual plots (bottom right).

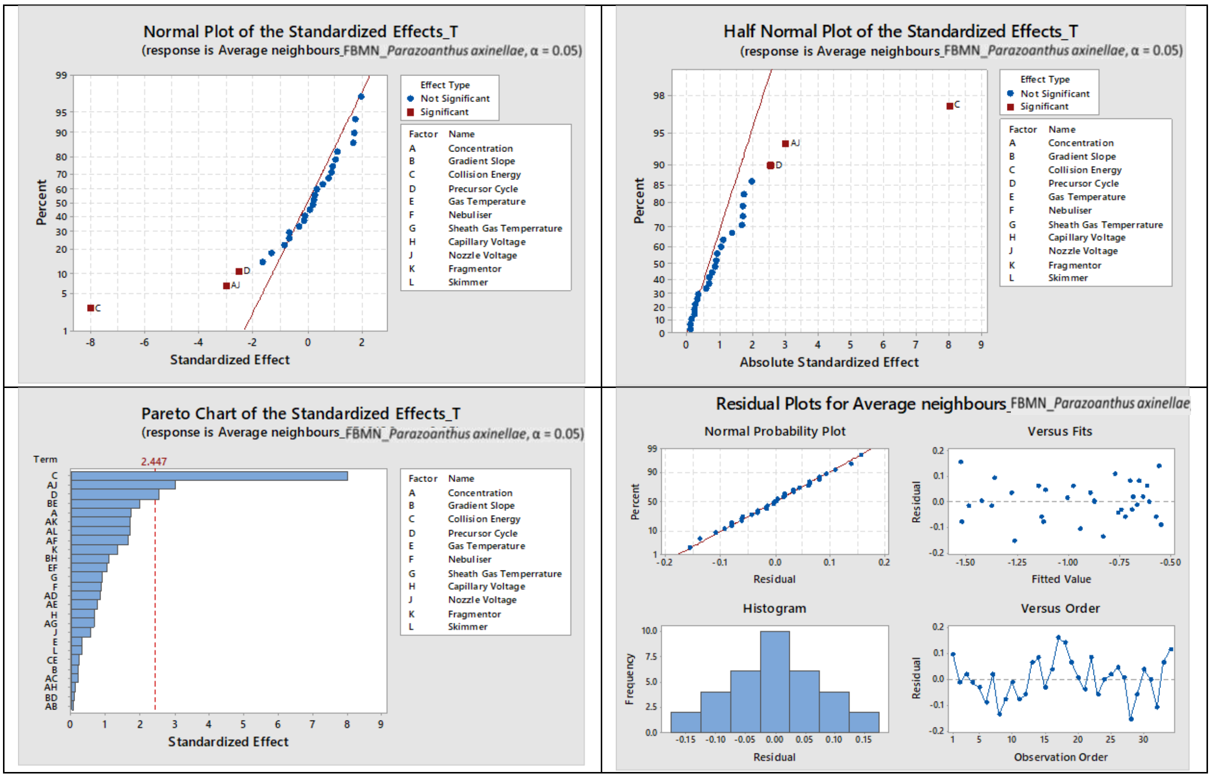

Figure S30: DOE reports for average number of neighbours model in Feature-Based Molecular Networking of *Parazoanthus axinellae*, normal plot of standardized effects (top left), half normal plot of standardized effects (top right), pareto chart of standardized effects (bottom left), and residual plots (bottom right).

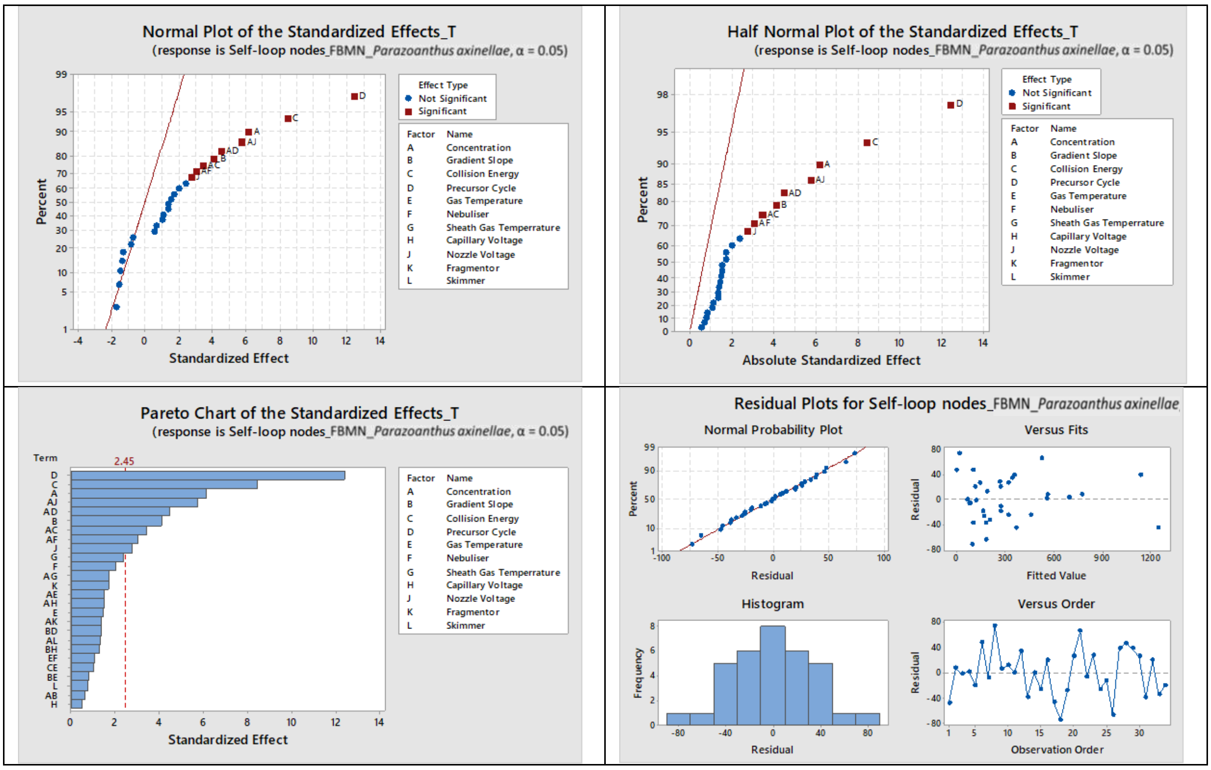

Figure S31: DOE reports for number of self-loop nodes model in Feature-Based Molecular Networking of *Parazoanthus axinellae*, normal plot of standardized effects (top left), half normal plot of standardized effects (top right), pareto chart of standardized effects (bottom left), and residual plots (bottom right).

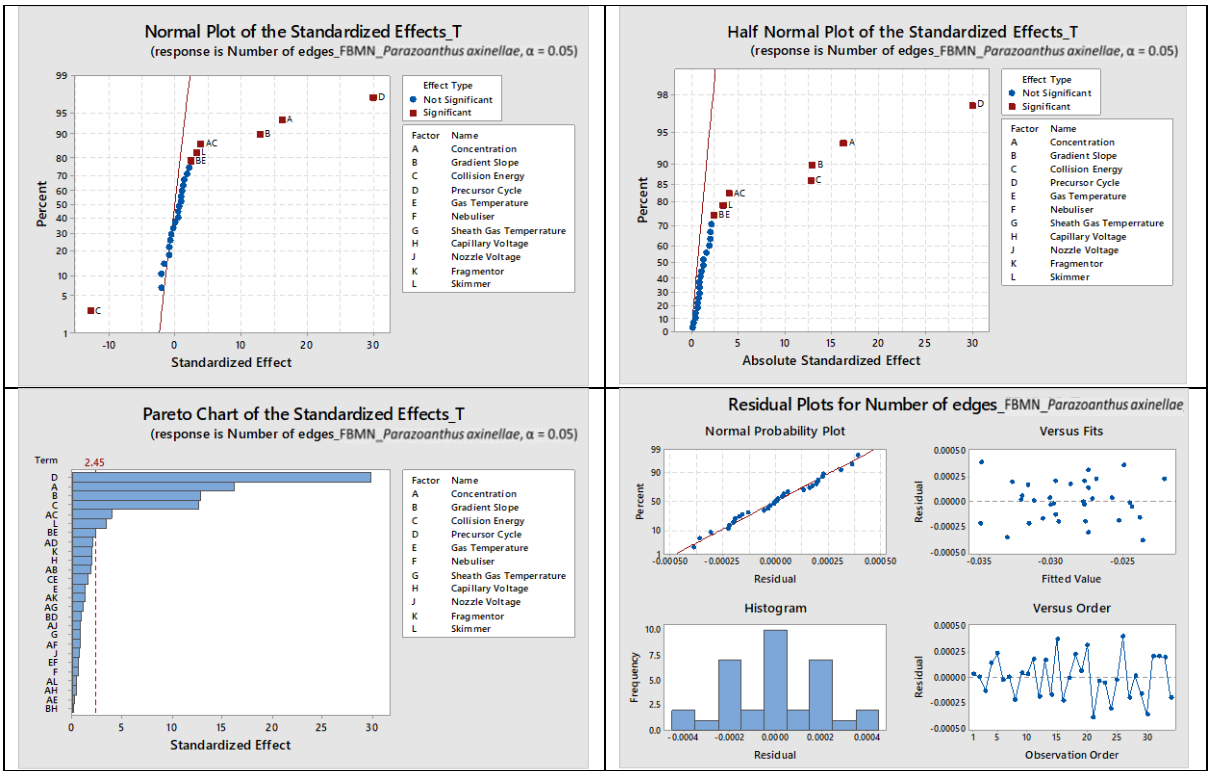

Figure S32: DOE reports for number of edges model in Feature-Based Molecular Networking of *Parazoanthus axinellae*, normal plot of standardized effects (top left), half normal plot of standardized effects (top right), pareto chart of standardized effects (bottom left), and residual plots (bottom right).

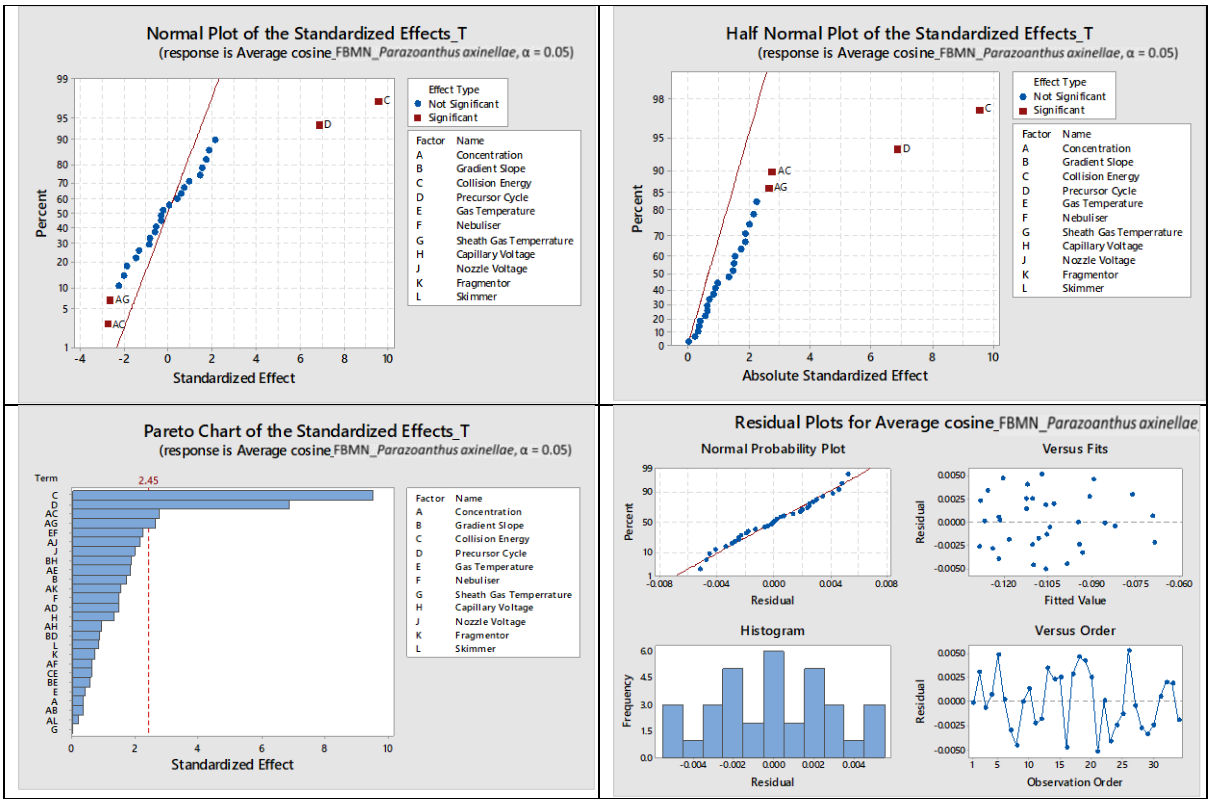

Figure S33: DOE reports for average cosine score model in Feature-Based Molecular Networking of *Parazoanthus axinellae*, normal plot of standardized effects (top left), half normal plot of standardized effects (top right), pareto chart of standardized effects (bottom left), and residual plots (bottom right).

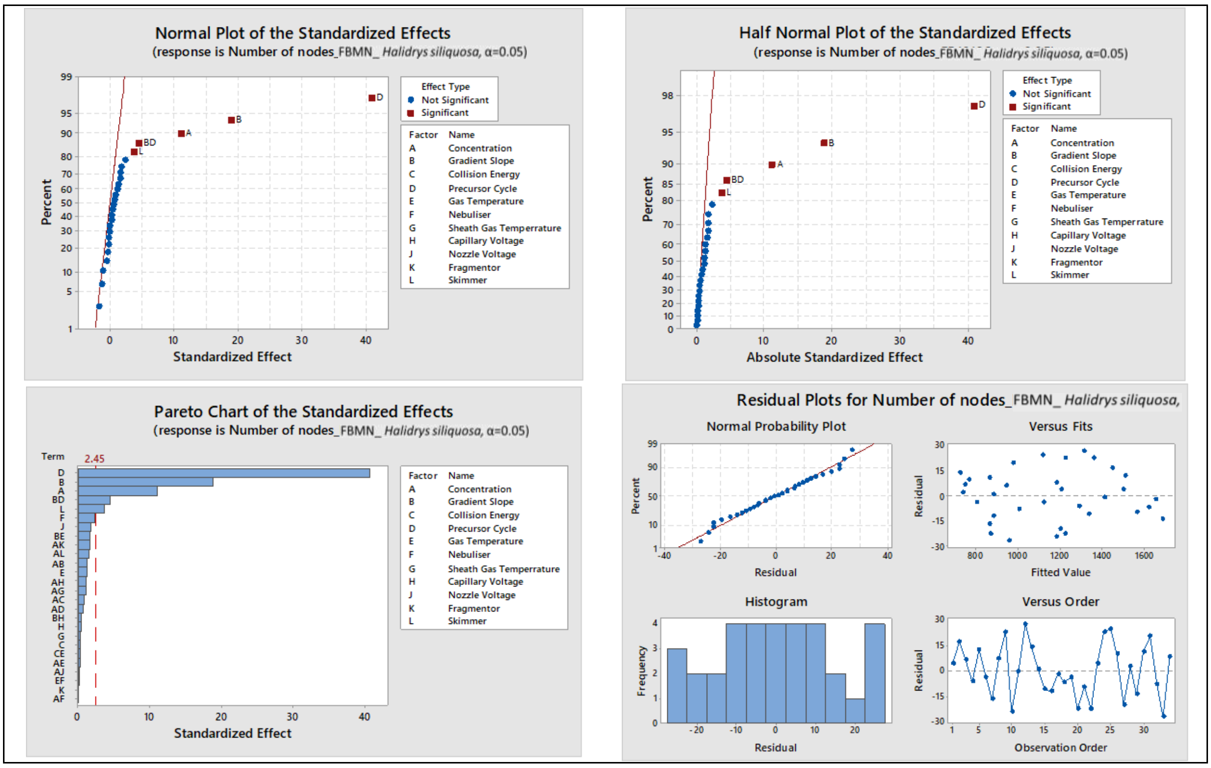

Figure S34: DOE reports for number of nodes model in Feature-Based Molecular Networking of *Halidrys siliquosa*, normal plot of standardized effects (top left), half normal plot of standardized effects (top right), pareto chart of standardized effects (bottom left), and residual plots (bottom right).

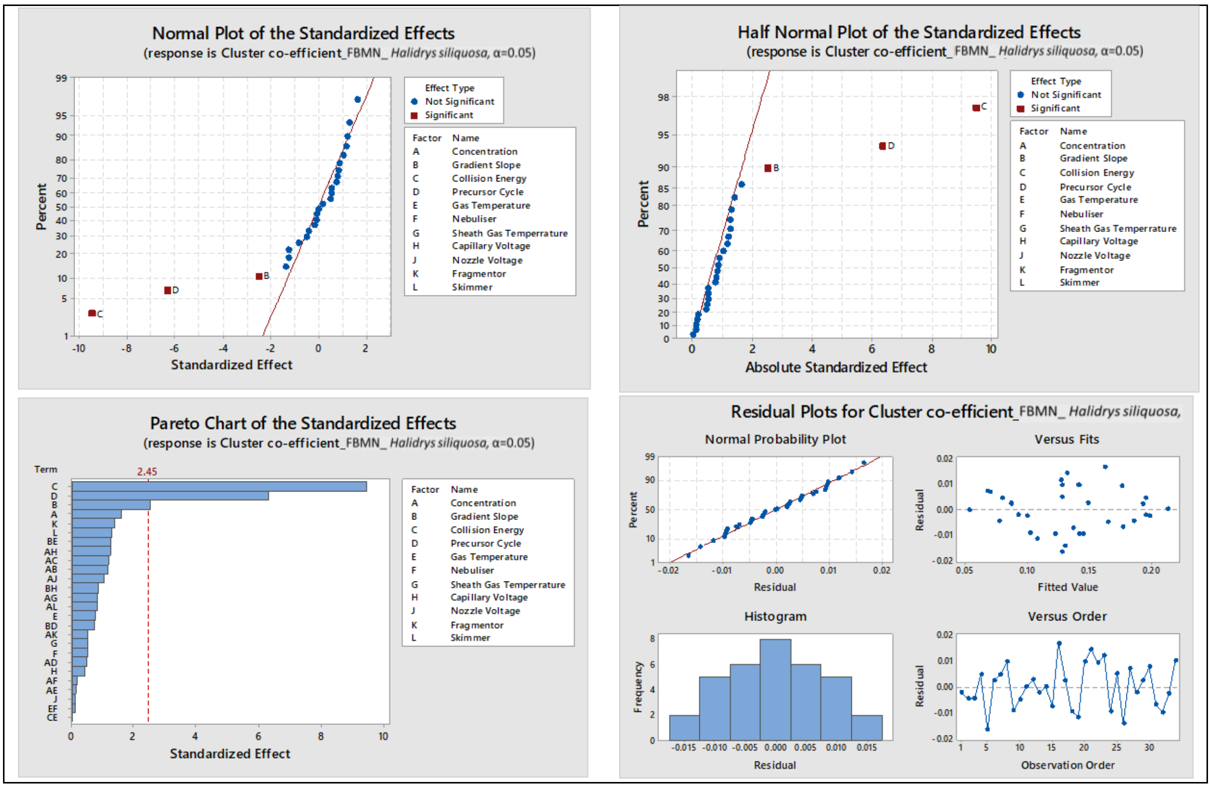

Figure S35: DOE reports for cluster co-efficient model in Feature-Based Molecular Networking of *Halidrys siliquosa*, normal plot of standardized effects (top left), half normal plot of standardized effects (top right), pareto chart of standardized effects (bottom left), and residual plots (bottom right).

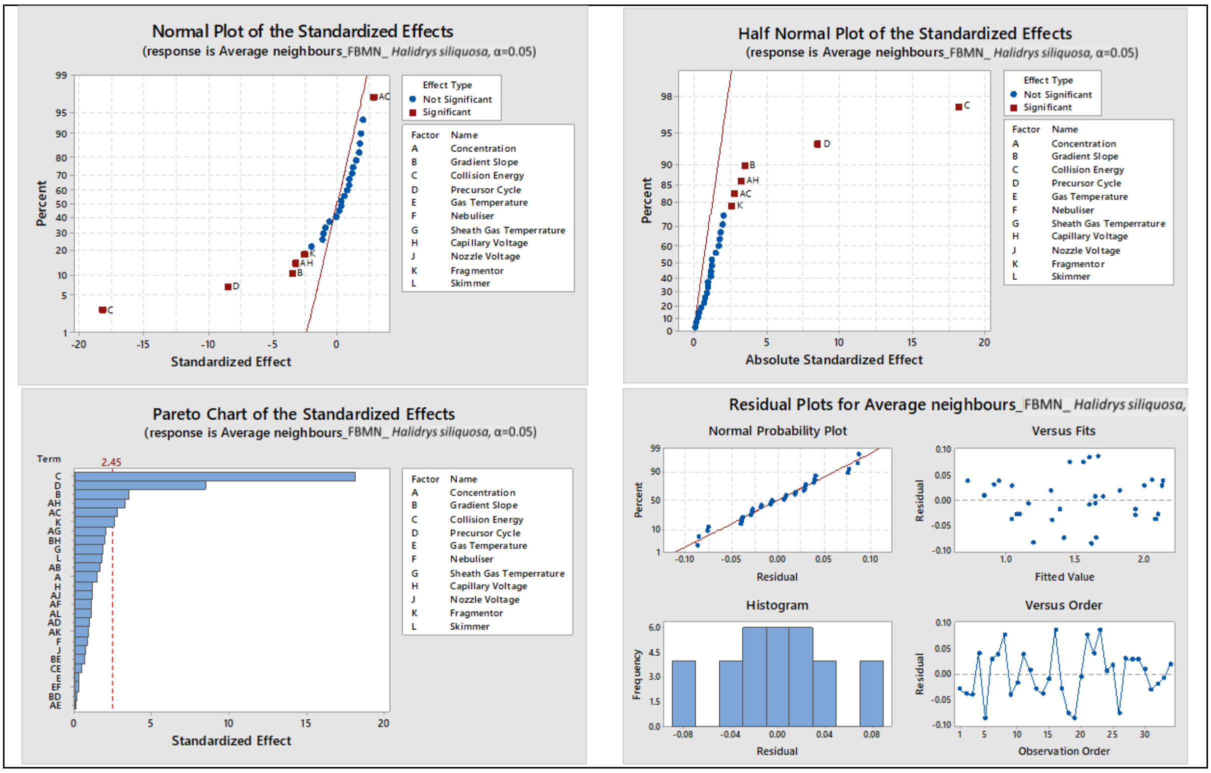

Figure S36: : DOE reports for cluster co-efficient model in Feature-Based Molecular Networking of *Halidrys siliquosa*, normal plot of standardized effects (top left), half normal plot of standardized effects (top right), pareto chart of standardized effects (bottom left), and residual plots (bottom right).

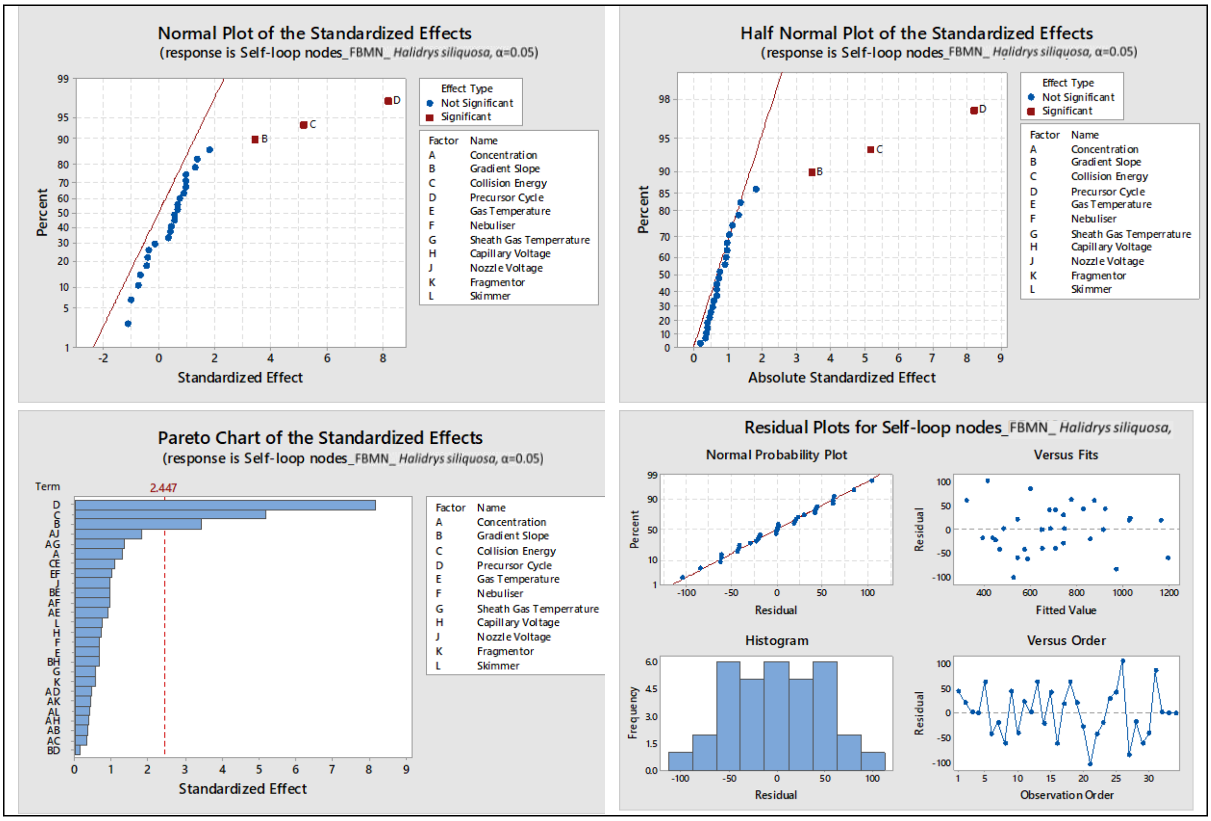

Figure S37: DOE reports for number of self-loop nodes model in Feature-Based Molecular Networking of *Halidrys siliquosa*, normal plot of standardized effects (top left), half normal plot of standardized effects (top right), pareto chart of standardized effects (bottom left), and residual plots (bottom right).

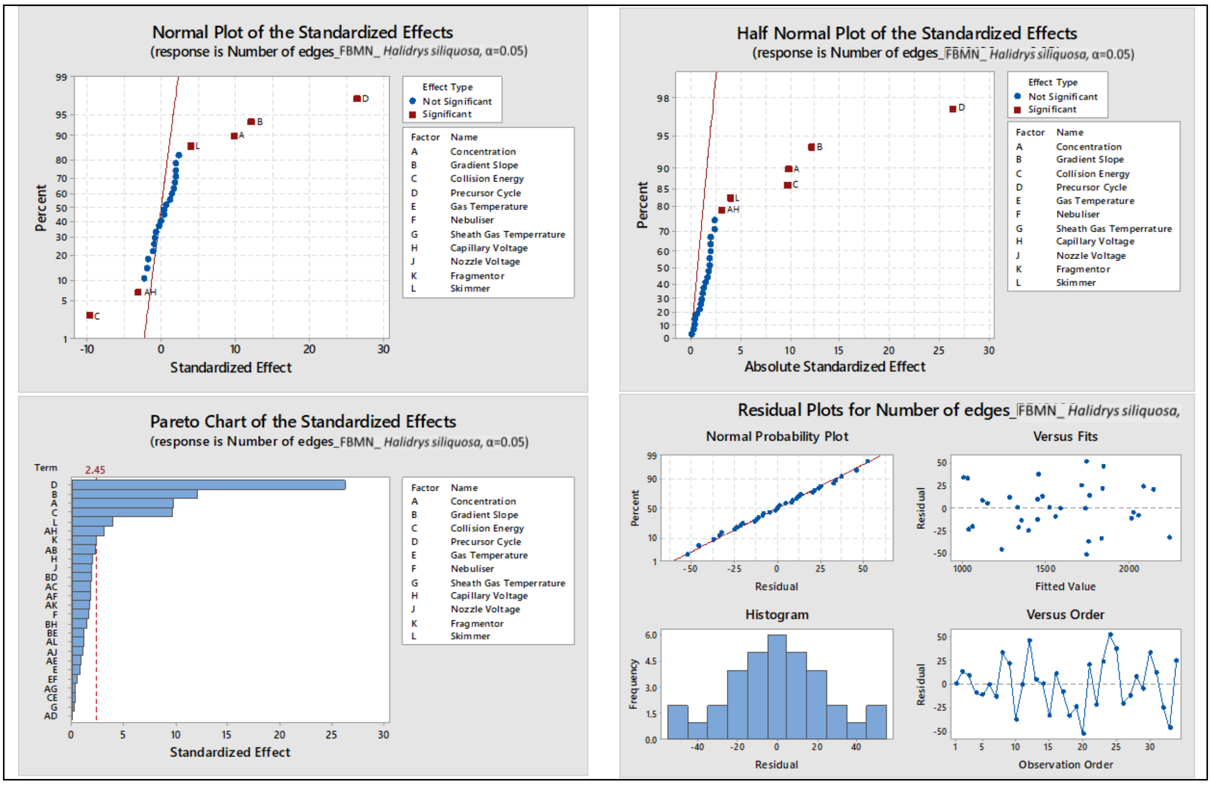

Figure S38: DOE reports for number of edges model in Feature-Based Molecular Networking of *Halidrys siliquosa*, normal plot of standardized effects (top left), half normal plot of standardized effects (top right), pareto chart of standardized effects (bottom left), and residual plots (bottom right).

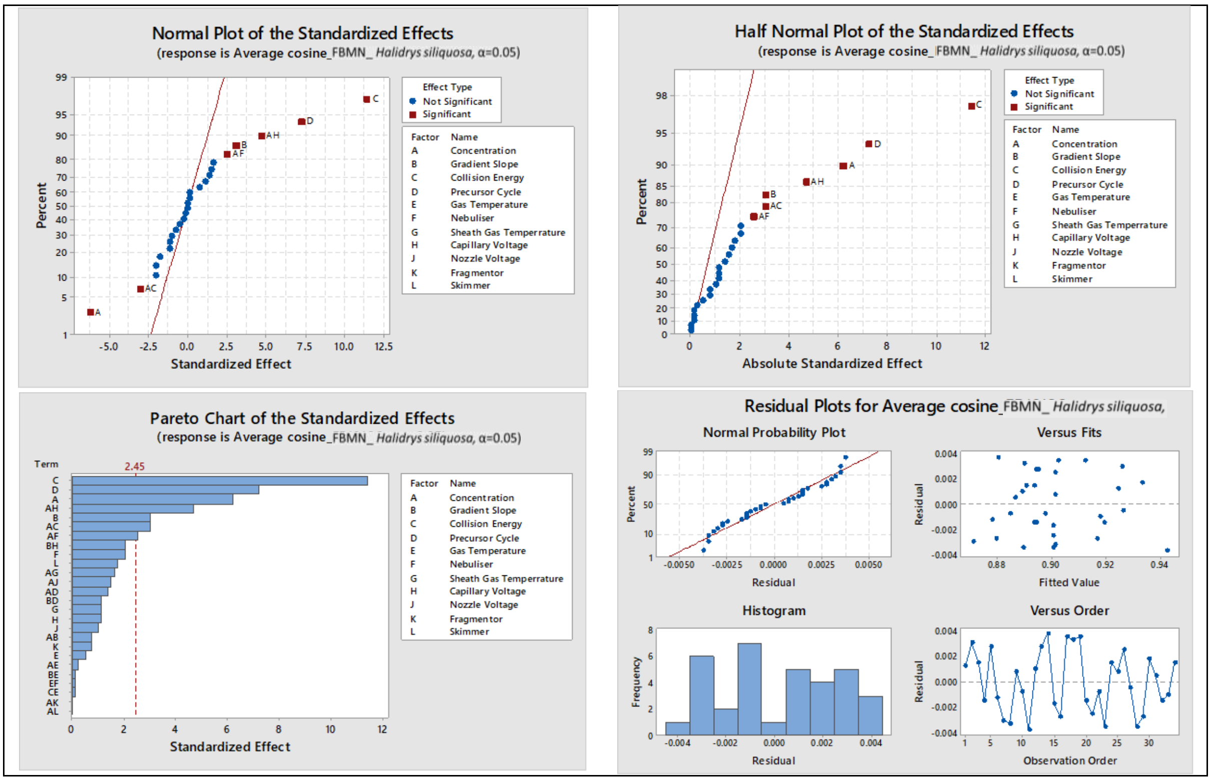

Figure S39: DOE reports for average cosine score model in Feature-Based Molecular Networking of *Halidrys siliquosa*, normal plot of standardized effects (top left), half normal plot of standardized effects (top right), pareto chart of standardized effects (bottom left), and residual plots (bottom right).
